# Systematic quantification of HDR and NHEJ reveals effects of locus, nuclease, and cell type on genome-editing

**DOI:** 10.1038/srep23549

**Published:** 2016-03-31

**Authors:** Yuichiro Miyaoka, Jennifer R. Berman, Samantha B. Cooper, Steven J. Mayerl, Amanda H. Chan, Bin Zhang, George A. Karlin-Neumann, Bruce R. Conklin

**Affiliations:** 1 Gladstone Institute of Cardiovascular Disease, San Francisco, California, 94158, USA; 2 Digital Biology Center, Bio-Rad Laboratories, Pleasanton, California, 94588, USA; 3 Departments of Medicine and Cellular and Molecular Pharmacology, University of California, San Francisco, California, 94143, USA

## Abstract

Precise genome-editing relies on the repair of sequence-specific nuclease-induced DNA nicking or double-strand breaks (DSBs) by homology-directed repair (HDR). However, nonhomologous end-joining (NHEJ), an error-prone repair, acts concurrently, reducing the rate of high-fidelity edits. The identification of genome-editing conditions that favor HDR over NHEJ has been hindered by the lack of a simple method to measure HDR and NHEJ directly and simultaneously at endogenous loci. To overcome this challenge, we developed a novel, rapid, digital PCR–based assay that can simultaneously detect one HDR or NHEJ event out of 1,000 copies of the genome. Using this assay, we systematically monitored genome-editing outcomes of CRISPR-associated protein 9 (Cas9), Cas9 nickases, catalytically dead Cas9 fused to FokI, and transcription activator–like effector nuclease at three disease-associated endogenous gene loci in HEK293T cells, HeLa cells, and human induced pluripotent stem cells. Although it is widely thought that NHEJ generally occurs more often than HDR, we found that more HDR than NHEJ was induced under multiple conditions. Surprisingly, the HDR/NHEJ ratios were highly dependent on gene locus, nuclease platform, and cell type. The new assay system, and our findings based on it, will enable mechanistic studies of genome-editing and help improve genome-editing technology.

Designer nucleases such as clustered, regularly interspaced, short palindromic repeats (CRISPR)-associated Cas9 are efficient genome-editing tools that hold great promise for experimental biology and therapies^1^,^2^,^3^. These tools induce a nick or a double-strand break (DSB) at targeted regions to activate two DNA repair pathways: homology-directed repair (HDR) and nonhomologous end-joining (NHEJ). HDR is a precise repair mechanism that uses homologous donor DNA to repair DNA damage, whereas NHEJ is an error-prone mechanism in which broken ends of DNA are joined together, often resulting in a heterogeneous pool of insertions and deletions. Recently, the targeting specificity of CRISPR-based systems was improved by the development of dual Cas9 D10A nickase (Cas9-D10A) and paired catalytically dead Cas9 fused to FokI (FokI-dCas9) systems^4^,^5^,^6^,^7^. Those different nuclease platforms, including another type of Cas9 nickases, Cas9 H840A nickase (Cas9-H840A), have different modes of DNA nicking or cleavage. Both TALENs and FokI-dCas9 rely on the two FokI nuclease domains, whereas the two catalytic sites of Cas9, RuvC (where D10 is located) and HNH (where H840 is located), are not equal and are clearly separated, as shown by biochemical and structural studies of Cas9-binding DNA^8^,^9^,^10^. Cas9-H840A cuts the noncomplementary DNA strand that is free of gRNA, whereas Cas9-D10A cuts the complementary strand that is hybridized with gRNA^1^,^11^. These differences may affect genome-editing outcomes, but there has been no systematic assessment for this possibility.

A major challenge for precise genome-editing is the ability to induce high-fidelity HDR edits with a low NHEJ background^12^,^13^. For example, in our attempts to isolate human induced pluripotent stem cell (iPSC) lines with genomic modifications via HDR, multiple isolated iPSC lines had one allele with desirable HDR and disruption of the other allele by NHEJ (Supplementary Table S1). These observations highlight the importance of minimizing the NHEJ activity to achieve precise genome-editing. However, owing to the limitations of detection methods, the activity of sequence-specific nucleases has been assessed mainly by detecting NHEJ^4^,^5^,^6^,^7^.

Methods to detect HDR and NHEJ rely on gel-based systems or artificial reporter assays—neither of which are suitable for systematic analysis of many editing conditions at endogenous gene loci^14^,^15^,^16^,^17^. The high resolution melting (HRM) curve analysis is a cost-effective assay to detect genome-editing outcomes, but its sensitivity or quantitative ability is limited^18^,^19^. Direct sequencing is an ideal method, but currently requires time and effort for library preparation and bioinformatics capability to analyze the data. Initial direct sequencing results suggest that HDR and NHEJ are activated differently by different nuclease platforms^16^. Thus, an assay is needed to quantify HDR and NHEJ simultaneously under many conditions. To meet this challenge, we adapted our highly sensitive droplet digital PCR (ddPCR) assay, which quantifies only HDR at endogenous loci^20^, to simultaneously measure both HDR and NHEJ. In this study, we systematically evaluated various genome-editing conditions with this ddPCR–based assay to identify those that preferentially induce HDR over NHEJ.

## Methods

### Statistical Information

For transfection experiments in HEK293T cells and HeLa cells, two different transfections were done in triplicate (total of 6 biological replicates). For experiments with human iPSC experiments, there were three different transfections (3 biological replicates). Two-tailed Student’s t-test was performed to address the difference between HDR and NHEJ-inducing activities of genome-editing conditions. No samples were excluded.

### Plasmids and Oligonucleotides

We used pX330 for wildtype Cas9 and pX335 for Cas9-D10A^3^,^4^. The H840A mutation was introduced into pX330 to create Cas9-H840A (pXCas9H840A, Addgene plasmid 60900). To compare FokI-dCas9 in the same plasmid backbone, we inserted FokI-dCas9 from pSQT1601^6^ into the plasmid backbone of pX330 (pXFokI-dCas9, Addgene plasmid 60901). ZiFiT was used to design the gRNAs for FokI-dCas9 (http://zifit.partners.org/ZiFiT/ ChoiceMenu.aspx)^21^,^22^ because FokI-dCas9 requires a specific spacer length between a pair of gRNAs^6^, whereas the dual Cas9 nickase system has a more relaxed rule for spacer length^4^ (Supplementary Table S2). ZiFiT was also used to design the TALENs, which were constructed with the Voytas laboratory’s Golden Gate assembly system, provided through Addgene (http://www.addgene.org/TALeffector/ goldengateV2/)^23^, except the backbone vector (Supplementary Table S3). We used the MR015 TALEN backbones^20^. Oligonucleotide donors were all 60 nt and had point mutations in the middle of them (Supplementary Table S4). Both sense and antisense strand oligonucleotide donors purified by standard desalting were purchased from Integrated DNA Technologies.

### HEK293T Cell and HeLa Cell Culture and Transfection

HEK293T cells and HeLa cells were maintained in Dulbecco’s modified Eagle medium with high glucose, sodium pyruvate, and L-glutamine (Life Technologies) supplemented with 10% FetalPlex (Gemini Bio-Products). For transfection, 4 × 10^4^ cells and 2 × 10^4^ cells were plated into each well of a 96-well plate for HEK293T cells and HeLa cells, respectively. One day later, the cells were transfected with DNA, using 0.3 μl of Lipofectamine 2000 (Life Technologies) per well, according to the manufacturer’s instructions. For transfections of single Cas9 systems, 90 ng of a plasmid for nucleases and 10 ng of oligonucleotide donor DNA were transfected per well. For dual-nuclease systems and TALENs, 45 ng of two vectors of nucleases and 10 ng of oligonucleotide donor DNA were transfected per well. Genomic DNA for *RBM20* and *GRN* was extracted from the cells 3 days after transfection as described^20^. Because the mutagenic efficiency was very low at the ATP7 locus, genomic DNA for *ATP7B* was extracted 6 days after transfection. Genomic DNA was resuspended in 30 μl of water per well. For transfection of HEK293T cells with Nucleofector, the same condition as for iPSCs described below except program Q-01 was used instead of A-23.

### Human iPSC Culture and Transfection

The protocol for iPSCs was approved by the Committee on Human Research of the University of California, San Francisco; approval no. 10-02521. The experimental procedures in this study were carried out in accordance with the approved guidelines. The human iPSC line used in this study was WTC11, which was generated from a healthy male patient^24^, using the episomal reprogramming method^25^. Informed consent was obtained from this iPSC line. The culture and transfection conditions are described elsewhere^20^. Briefly, for transfection, 6 μg of vector for single nuclease systems or 3 μg of each vector for dual-nuclease systems, and 6 μg of an oligonucleotide donor DNA was transfected into 2 million iPSCs with the Human Stem Cell Nucleofector Kit-1 and a Nucleofector 2b (Lonza) using program A-23. One sixth of the transfected cells were plated in each well of a 6-well plate. Four days after transfection, genomic DNA from the cells was purified with the DNeasy Blood & Tissue Kit (Qiagen).

### Probes, Primers, and Synthetic Alleles for ddPCR Assay

All ddPCR assays were designed with Primer3Plus (http://primer3plus.com) with modified settings compatible with the master mix: 50 mM monovalent cations, 3.0 mM divalent cations, and 0 mM dNTPs with SantaLucia 1998 thermodynamic and salt correction parameters. Predicted nuclease cut sites (3 base pairs upstream of PAM for CRISPR, equidistant between DNA binding domains for TALEN or FokI-dCas9) were positioned mid-amplicon, with 75–125 base pairs flanking either side up to the primer binding sites. To ensure quantification of integrated edits, at least one primer was positioned outside the donor molecule sequence. Reference probe and primers were designed distant from the cut site (and nexus of NHEJ generation) to avoid loss of binding sites. Optimal annealing temperature was determined empirically with a temperature gradient. In some cases, a dark, nonextendible oligonucleotide (3′ phosphorylation) was designed to block cross-reactivity of the HDR probe and the WT sequence. Probe position and number varied depending on the relative positions of the cut site(s) and edit site (Supplementary Fig. S1 and Supplementary Tables S5 and S6).

Synthetic double-stranded DNA controls were manufactured as positive controls for assay validation (gBlocks, Integrated DNA Technologies) (Supplementary Table S7). HDR-positive controls contained the point mutation at the desired edit site; NHEJ-positive controls had a 1-base pair deletion at the predicted nuclease cut site. Synthetic NHEJ insertion controls performed comparably to the 1-base pair deletion controls (Supplementary Fig. S2). Lyophilized gBlocks were resuspended in 250 μl of TE + 100 ng/μl polyA carrier (Roche, Catalog no. 10108626001). Two additional 200-fold dilutions in TE+polyA resulted in a master stock of around 40,000 copies/μl that was maintained in LoBind tubes (Eppendorf) and confirmed by ddPCR quantification. Master high-copy gBlock stocks were kept in a post-PCR environment to avoid contamination.

### ddPCR to Detect HDR and NHEJ

The 20× ddPCR assay premixtures were composed of forward and reverse primers (18 μM each), reference probe (5 μM), HDR probe (5 μM), NHEJ probes (5 μM), and a dark probe (10 μM), depending on the assay (Supplementary Table S6). We mixed the following reagents in a 96-well plate to make a 25-μl reaction: 12.5 μl of ddPCR Supermix for Probes (no dUTP) (Bio-Rad Laboratories #186-3024), 1.25 μl of 20x assay, 10 U of HindIII-HF (for *RBM20* and *ATP7B*, New England BioLabs #R3104S) or 5 U of MseI (for *GRN*, New England BioLabs #R0525S), 100–150 ng of genomic DNA in water, and water up to 25 μl. Droplets were generated with 20 μl of the premixed reaction and a QX100 Droplet Generator according to the manufacturer’s instructions (Bio-Rad Laboratories) and transferred to a 96-well PCR plate for standard PCR on a C1000 Thermal Cycler with a deep well block (Bio-Rad Laboratories).

Two thermal cycling programs were used: (1) step 1, 95 °C for 10 min; step 2, 94 °C for 30 s; step 3, 59 °C for 1 min; repeat steps 2 and 3 39 times; step 4, 98 °C for 10 min with all the steps ramped by 2 °C/s (for *RBM20*) and (2) step 1, 95 °C for 10 min; step 2, 94 °C for 30 s; step 3, 58 °C for 1 min; step 4, 72 °C for 2 min; repeat steps 2, 3, and 4 39 times; step 5, 98 °C for 10 min with all steps ramped by 2 °C/s (for *GRN* and *ATP7B*).

After PCR, the droplets were analyzed with a QX100 Droplet Reader (Bio-Rad Laboratories) with the “absolute quantification” option. All the samples were analyzed with genomic DNA samples without modification (negative control) or with known modification by Cas9 (positive control) to determine the proper gating for HDR and NHEJ events. In two-dimensional plots, droplets without templates were gated as a black population, while all droplets positive for HDR (FAM++) were gated as an orange population. Droplets containing only NHEJ alleles (FAM+ HEX−) were gated as a blue population. All other droplets were gated as a green population. For some assays with multiple NHEJ probes, the NHEJ cluster does not entirely lose HEX fluorescence (Supplementary Figs S1 and S4). To analyze enough number of genomic DNA copies to detect one HDR or NHEJ event out of 1,000 copies, we adjusted the genomic DNA concentration to 100–2,000 copies/μl (2,000–40,000 copies per reaction). The allelic frequencies of unmodified, HDR, and NHEJ alleles were quantified as described below.

### Quantification of ddPCR data

Two-dimensional droplet cluster plots were thresholded as described above (Supplementary Fig. S4).

The standard formula for ddPCR quantification is:


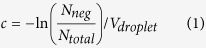


where

*N*_*neg*_ = the number of droplets that do not contain the species of interest.

*N*_*total*_ = the total number of droplets

*V*_*droplet*_ = the volume of an analyzed sample

For the assay described here, some droplet populations cannot easily be separated, such as the droplet group containing WT and NHEJ+WT droplets (green) and the droplet group containing HDR and HDR+WT droplets (orange). In those cases, an appropriate subset of droplets was used to calculate N_neg_ and N_total_.

### Definitions

- N_empty_ = number of droplets in the double-negative cluster labeled “empty” (black).
- N_NHEJ_ = number of droplets in the cluster labeled “NHEJ” (blue).
- N_WT+_ = number of droplets in the clusters labeled “WT+” (green).
- N_HDR+_ = number of droplets in the clusters labeled “HDR+” (orange).

For NHEJ quantification, NHEJ single-positive droplets and the Empty (double-negative) droplets were used. For WT quantification, NHEJ, Empty, and WT droplets were used. For HDR quantification, all droplets were used.

### For NHEJ Quantification

- (2) N_neg_ = N_empty_
- (3) N_total_ = N_empty_ + N_NHEJ_

### For HDR Quantification

- (4) N_neg_ = N_empty_ + N_NHEJ_ + N_WT+_
- (5) N_total_ = N_empty_ + N_NHEJ_ + N_WT+_ + N_HDR+_

### For WT Quantification

- (6) N_neg_ = N_empty_ + N_NHEJ_
- (7) N_total_ = N_empty_ + N_NHEJ_ + N_WT+_

## Results

### Assay System to Simultaneously Detect HDR and NHEJ

**Figure 1:**
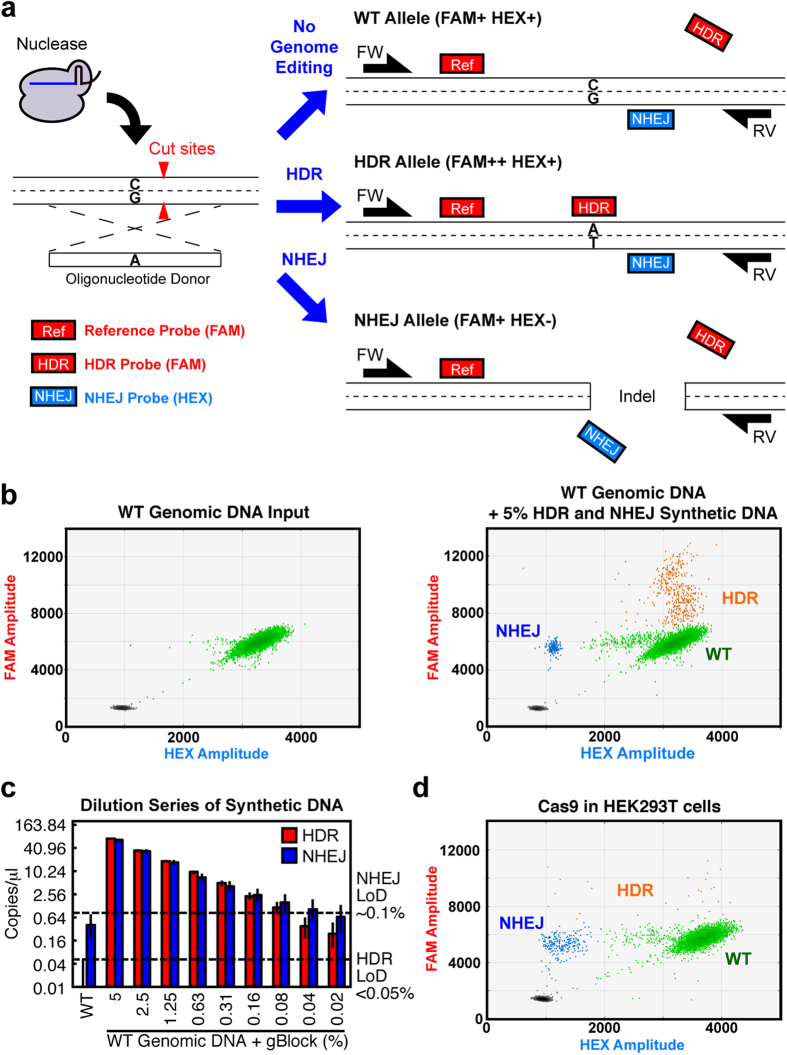
Design and validation of the assay to simultaneously detect HDR and NHEJ at an endogenous gene locus. (**a**) The WT allele will be FAM+ and HEX+ because the reference (FAM) and NHEJ (HEX) probes bind to it. With HDR, the HDR probe can bind to the HDR allele, making it FAM++ (higher-amplitude fluorescence than FAM+). With NHEJ, the NHEJ probe loses its binding site, so the NHEJ allele will be FAM+ and HEX−. (**b**) Validation of the assay with synthetic DNA spiked into WT genomic DNA. Analysis of unspiked WT genomic DNA without genome-editing showed only the FAM+ and HEX+ WT (green) allele on the left 2D droplet scatter plot. The assay robustly detected a spike-in of 5% of synthetic HDR (orange) and NHEJ (blue) alleles added to the genomic DNA. (**c**) Assay sensitivity established by 2-fold serial dilution of HDR and NHEJ synthetic template in a constant (100 ng) background of WT genomic DNA. The limit of detection (LoD) was ~0.1% for NHEJ and <0.05% for HDR, as established by comparison with WT genomic DNA-only wells (nonoverlap of 95% confidence intervals, dotted line). Data represent two merged wells per dilution point and four merged wells for WT genomic DNA-only negative control. The 95% confidence interval is shown. (**d**) Simultaneous detection of HDR (0.6%) and NHEJ (5.3%) induced by Cas9 in HEK293T cells.

We designed three kinds of probes, all residing within one amplicon. The first, a reference probe (FAM) located away from the mutagenesis site, counts all genomic copies of the target. The second, an NHEJ probe (HEX) located where nucleases cut or nick genomic DNA, has a wildtype (WT) sequence. If nucleases induce NHEJ, the NHEJ probe loses its binding site, resulting in loss of HEX and leaving only the FAM signal of the reference probe. This is similar to a strategy that has previously been used to detect multiple mutations, insertions, and deletions^26^,^27^. The third probe (also FAM) binds the desired HDR point-mutation site, causing a gain of additional FAM signal when precise edits are present (Fig. 1a and Supplementary Fig. S1 and Supplementary Tables S5 and S6).

We designed the initial assay for point mutagenesis in *RBM20*, a gene important in hereditary cardiomyopathy^28^. To validate probes and primers, we used synthetic DNA representing the HDR and NHEJ (1-base pair deletion) alleles (Supplementary Table S7). We analyzed WT genomic DNA spiked with different amounts of the synthetic alleles and found that the assay was reproducible and linear over a wide range of input DNA. With this strategy, NHEJ (limit of detection ~0.1%) and HDR (limit of detection <0.05%) were detected as distinct, spatially segregated NHEJ- and HDR-positive droplets with low background signal (Fig. 1b,c and Supplementary Fig. S2). This demonstrates that one HDR or NHEJ event from 1,000 copies of the genome can be captured by this assay. Synthetic NHEJ alleles containing either a 1-base pair deletion, a 1-base pair insertion, or a 4-base pair insertion were readily detected, demonstrating that this assay detects a variety of insertions or deletions created by NHEJ (Supplementary Fig. S2). When applied to Cas9-mediated mutagenesis at RBM20 in HEK293T cells, the assay detected 79, 677, and 11,971 copies of the HDR, NHEJ, and WT alleles, respectively (0.6% HDR and 5.3% NHEJ) in a non-clonal pool of edited cells. Thus, the assay sensitively and precisely detected HDR and NHEJ in genomic DNA from edited cells (Fig. 1d).

### Systematic Quantification of HDR and NHEJ Alleles Generated by Diverse Editing Strategies

**Figure 2:**
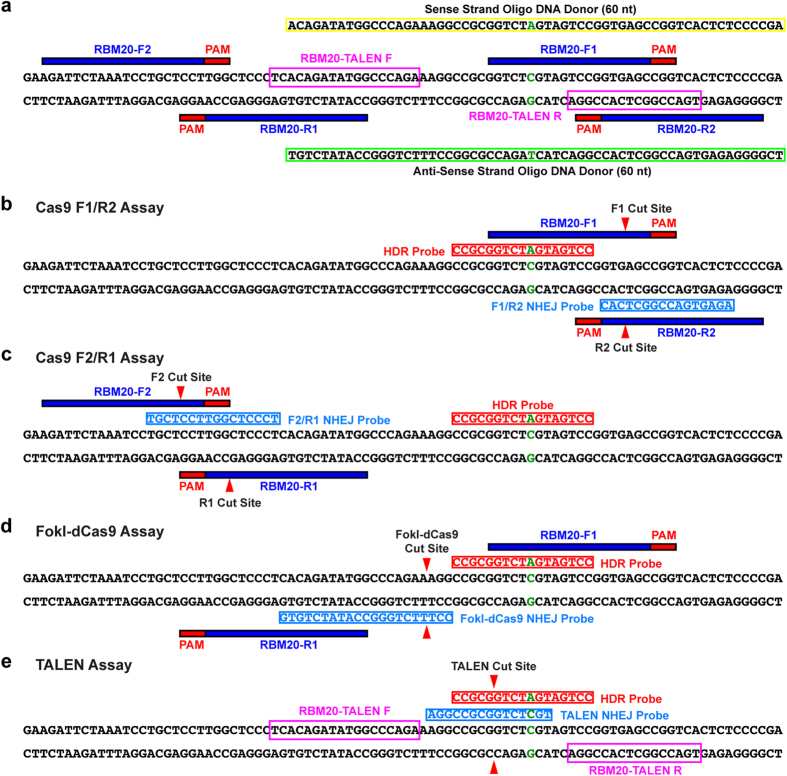
Design of point mutagenesis and assay systems for RBM20. (**a**) Design of point mutagenesis for RBM20. The locations of gRNAs (F1, F2, R1, or R2), TALENs, and donor DNAs are shown. The mutation sites are highlighted in green. The locations and sequences of sense and antisense strand oligonucleotide donors (60 nt) are also shown. (**b–e**) Design of assay systems for RBM20. The locations of HDR and NHEJ probes are shown for the Cas9 with gRNA-F1/R2 (**b**), Cas9 with gRNA-F2/R1 (**c**), FokI-dCas9 (**d**), and TALEN (**e**) assays. Note that two NHEJ probes were included in dual Cas9 assays (see Supplementary Fig. S1 and Supplementary Table S6). For simplicity, primers and reference probes are not included here (see Supplementary Table S5 for their sequences). Red triangles indicate the predicted cut sites by nucleases. HDR probes specifically bind to alleles induced by HDR, whereas NHEJ probes lose their binding sites when insertions or deletions are created by NHEJ.

To identify conditions that maximize HDR and minimize NHEJ, we next used our assay strategy to investigate genome-editing conditions at three human disease loci—*RBM20, GRN*, and *ATP7B* (Supplementary Table S8). We modulated the design, combination, and orientation of guide RNA (gRNA) as well as the nuclease type, 60 nt donor oligonucleotide orientation, and cell type. At each locus, we designed four gRNAs (F1, F2, R1, and R2) for CRISPR experiments and a pair of transcription activator–like effector nucleases (TALENs) to induce disease-related point mutations. F1 and F2 gRNAs are on sense strands; R1 and R2 gRNAs are on antisense strands. F1 gRNA covers the mutation site in the three genes; F2 gRNA does not (Fig. 2a, and Supplementary Figs S3 and S5, and Supplementary Tables S2–S4). We also designed HDR probes to capture point mutations, and NHEJ probes to detect insertions and deletions for individual nuclease types depending on their predicted cut sites (Fig. 2b–e and Supplementary Tables S5–S7). Equal amounts (input, 2,000–40,000 copies) of unmodified control and edited genomic DNA samples were examined for each assay to identify positive signals from editing (Supplementary Fig. S6).

### Single and Dual Cas9 Nickase Systems Induce More HDR than NHEJ in HEK293T Cells

**Figure 3:**
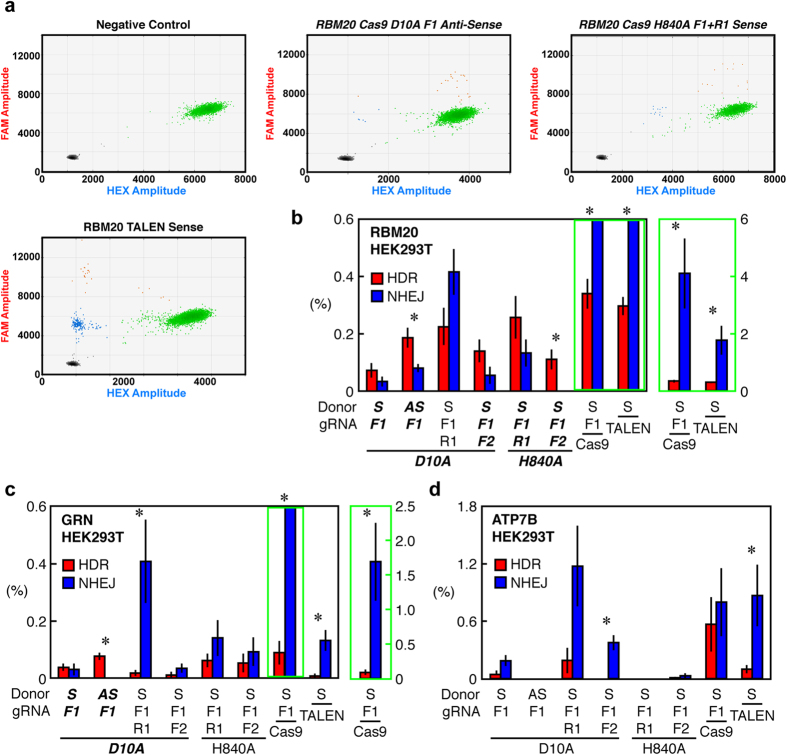
Measurement of HDR and NHEJ induced by sequence-specific nucleases in HEK293T cells. (**a**) Scatter plots of droplets showing HDR- and NHEJ-inducing activities of nucleases at *RBM20*. Representative 2D scatter plots of droplets positive for HDR (orange), NHEJ (blue), WT (green) alleles were obtained from HEK293T cells transfected with indicated nucleases targeting *RBM20*. Unedited WT genomic DNA was used as a negative control. Conditions that gave equivalent or more HDR than NHEJ are highlighted in bold italic. (**b–d**) Quantified genome-editing outcomes at *RBM20* (**b**), *GRN* (**c**), and *ATP7B* (**d**) in HEK293T cells. HDR (red) and NHEJ (blue) allelic frequency (%) is shown. Conditions that gave equivalent or more HDR than NHEJ are highlighted in bold italic. Values are mean ± SEM. (n = 6). The difference between the HDR and NHEJ frequencies were evaluated by Student’s T-test (*p < 0.05). S, sense strand oligonucleotide donor; AS, antisense strand oligonucleotide donor. The background signals of assays from equivalent amounts of unedited WT genomic DNA were subtracted from edited genomic DNAs (Supplementary Fig. S6).

First, we tested single Cas9 nickases (Cas9-D10A and Cas9-H840A) individually in HEK293T cells. Only Cas9-D10A induced more HDR than NHEJ, especially with antisense strand donor DNA at RBM20 (0.2% HDR with 0.1% NHEJ) and GRN (0.1% HDR, undetectable NHEJ) (Fig. 3a–c and Supplementary Fig. S7).

We next tested dual-nickase systems. The widely used dual Cas9-D10A platform has two gRNAs with their protospacer adjacent motifs facing outward (F1 and R1 gRNAs) (Fig. 2a and Supplementary Figs S3 and S5)^4^,^5^. With this configuration, dual Cas9-D10A induced at least twofold more NHEJ than HDR at all three loci. Surprisingly, dual Cas9-H840A with F1 and R1 gRNAs induced more HDR (0.2%) than NHEJ (0.1%) at *RBM20*, primarily by inducing less NHEJ than Cas9-D10A (Fig. 3a,b and Supplementary Fig. S7). The HDR:NHEJ ratio at *GRN* was also greater with dual Cas9-H840A than dual Cas9-D10A (Fig. 3c and Supplementary Fig. S7). However, with these same gRNA configurations, only dual Cas9-D10A had measurable genome-editing activity at *ATP7B*, mostly NHEJ (Fig. 3d and Supplementary Fig. S7). These results show that more HDR than NHEJ can be achieved by single and dual Cas9 nickase systems, but such conditions are highly dependent on the gene locus or location in the genome.

### Tandem Nicking by Cas9 Nickase Can Induce More HDR than NHEJ in HEK293T Cells

We further explored the effect of combination and strand orientation of gRNAs on editing by paired nickases. Using Cas9-D10A and Cas9-H840A with two gRNAs on the sense stand (F1 and F2 gRNAs) to nick the same strand (“tandem nicking”) (Supplementary Fig. S5) induced more HDR than NHEJ at *RBM20*. Tandem nicking by Cas9-H840A with F1 and F2 gRNAs induced 0.1% HDR and undetectable NHEJ at *RBM20* (Fig. 3b and Supplementary Fig. S7). However, the HDR:NHEJ ratios were lower at *GRN* and *ATP7B* (Fig. 3c,d and Supplementary Fig. S7). Thus, both sequence and gRNA combination influence the outcome of dual nickase editing.

In general, FokI-dCas9 and TALENs induced more NHEJ than HDR in HEK293T cells, but TALENs had higher overall activity than FokI-dCas9 (Fig. 3 and Supplementary Fig. S7). Cas9 had very high NHEJ-inducing activity (>10-fold higher than HDR) at *RBM20* and *GRN* yet strikingly was the best HDR inducer at *ATP7B* (0.6% HDR, 0.8% NHEJ) (Fig. 3). We also found that although the overall trend of NHEJ-inducing activity was the same in the presence and absence of donor DNA, its activity was generally greater in the presence of donor DNA in HEK293T cells (Supplementary Fig. S8). Thus, genome-editing outcomes are dependent on gene locus or genomic location, can be modulated by the nuclease system, and are influenced by the position and combination of editing components.

### Genome-Editing Outcome Is Dependent on Cell Type

**Figure 4:**
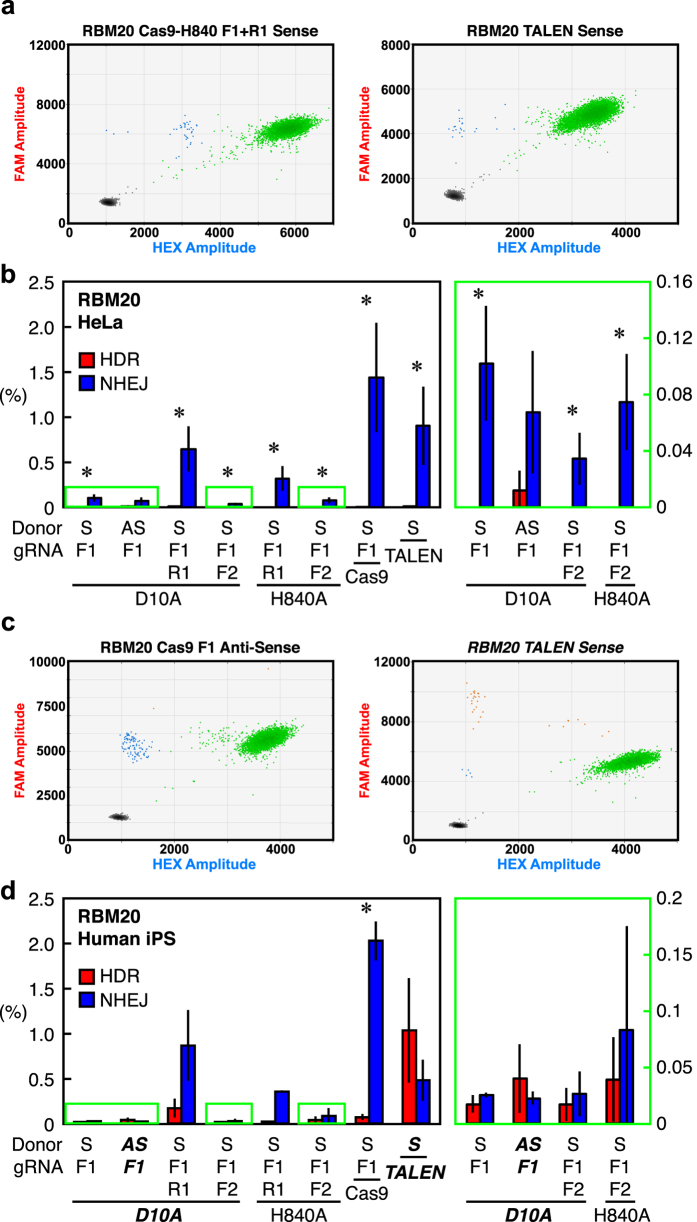
Measurement of HDR and NHEJ induced by sequence-specific nucleases at RBM20 in HeLa cells and iPSCs. (**a**) Scatter plots of droplets showing HDR- and NHEJ-inducing activities of dual Cas9-H840A and TALEN at *RBM20*. Representative 2D scatter plots of droplets positive for NHEJ (blue) and WT (green) alleles were obtained from HeLa cells transfected with dual Cas9-H840A or TALENs targeting *RBM20*. Both systems failed to induce HDR. (**b**) Quantified genome-editing outcomes at *RBM20* in HeLa cells. HDR (red) and NHEJ (blue) allelic frequency (%) is shown. (**c**) Scatter plots of droplets showing HDR- and NHEJ-inducing activities of Cas9 and TALEN at *RBM20*. Representative 2D scatter plots of droplets positive for HDR (orange), NHEJ (blue), WT (green) alleles were obtained from human iPSCs transfected with Cas9 or TALENs targeting *RBM20*. Only TALENs induced more HDR than NHEJ (highlighted in bold italic). (**d**) Quantified genome-editing outcomes at *RBM20* in iPSCs. HDR (red) and NHEJ (blue) allelic frequency (%) is shown. Conditions that gave equivalent or more HDR than NHEJ are highlighted in bold italic. For (**b**,**d**), values are mean ± SEM. (n = 6). The difference between the HDR and NHEJ frequencies were evaluated by Student’s T-test (*p < 0.05). S, sense strand oligonucleotide donor; AS, antisense strand oligonucleotide donor. The background signals of assays from equivalent amounts of unedited WT genomic DNA were subtracted from edited genomic DNAs (Supplementary Fig. S6). Conditions with low activity are shown in a different scale (highlighted in green).

To test the effect of cell type difference on genome editing, we transfected HeLa cells with the same single and dual Cas9 nickases, single Cas9, FokI-dCas9, and TALENs for *RBM20, GRN*, and *ATP7B* tested in HEK293T cells. We found that the frequency of HDR is very low compared to NHEJ in any conditions we tested in HeLa cells, demonstrating a clear cell type dependency of genome-editing outcomes (Fig. 4a,b, and Supplementary Fig. S9).

Next, we investigated genome-editing at *RBM20* in human iPSCs. Even though we used the same nucleases, their activities differed drastically from those in HEK293T cells and HeLa cells. The Cas9-based platforms had little activity or predominantly NHEJ-inducing activity (<0.2% HDR) in iPSCs. Conversely, TALENs induced 1% HDR with 0.5% NHEJ—the opposite of the pattern obtained with the same TALEN set in HEK293T cells and HeLa cells (Fig. 4c,d, and Supplementary Fig. S10). We also confirmed that the observed cell-type dependency was not due to the different transfection methods for HEK293T cells and iPSCs (Supplementary Fig. S11). These results reveal that genome-editing outcomes are dependent on cell type, as well as on gene locus and nuclease platform.

### NHEJ Allele Frequency Does Not Correlate with HDR Allele Frequency

**Figure 5:**
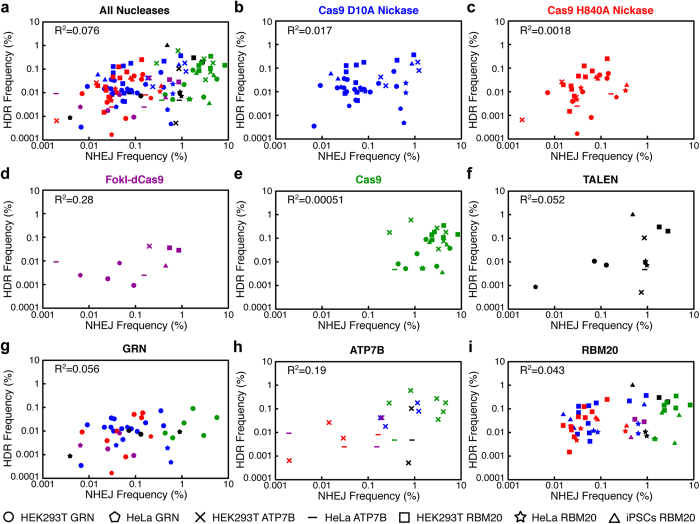
Little correlation between HDR and NHEJ frequencies induced by genome-editing. Scatter plots of HDR- and NHEJ-inducing activities of genome-editing conditions. (**a–i**) The frequency of HDR and NHEJ induced by all tested conditions (**a**), by Cas9-D10A (**b**) (blue), by Cas9-H840A (**c**) (red), by FokI-dCas9 (**d**) (purple), by Cas9 (**e**) (green), by TALEN (**f**) (black), at *GRN* (**g**), at *ATP7B* (**h**), and at *RBM20* (**i**) are plotted. The raw data are shown in Figs 3 and 4 and Supplementary Figs S7,S9, and S10. The shapes of the data points represent *GRN* in HEK293T cells (circle) or HeLa cells (pentagon), *ATP7B* in HEK293T cells (cross) or HeLa cells (bar), and *RBM20* in HEK293T cells (square), HeLa cells (star) or human iPSCs (triangle). The R^2^ values for the plots are shown. There is little correlation between HDR and NHEJ in general. Note that data points with the 0 value are not shown in the plots but were included for calculation of the R^2^ values.

**Figure 6:**
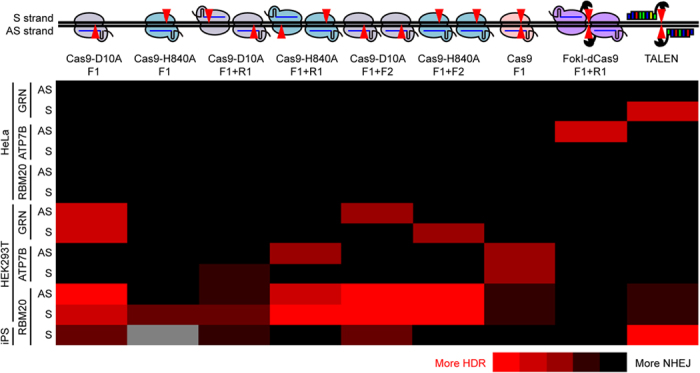
Schematic of nuclease-induced genome modifications and heat map of HDR-inducing activity. The HDR- and NHEJ-inducing activities of tested conditions are summarized as a heat map. The raw data are shown in Figs 3 and 4 and Supplementary Figs S7, S9 and S10. The conditions from the best (red) to the worst (black): >0.1% HDR and HDR>NHEJ, <0.1% HDR and HDR>NHEJ, 2× HDR>NHEJ>1× HDR, >0.1% HDR and 2× HDR<NHEJ, and <0.1% HDR and 2× HDR<NHEJ. Single Cas9-H840A was not tested in iPSCs (gray).

Because of limitations in detection methods, previous work has used NHEJ frequency as a surrogate for predicting rates of HDR^4^,^6^,^7^,^29^. We investigated the potential relationship between NHEJ and HDR frequency induced by genome-editing tools. HDR and NHEJ frequency did not correlate overall (R^2^ = 0.076) (Fig. 5a) or for individual nuclease platforms or gene loci (R^2^ = 0.00051~0.28) (Fig. 5b–i). These results indicate that the NHEJ-inducing activity does not always reflect the HDR-inducing activity. Overall, our findings show that the HDR:NHEJ ratio induced by genome-editing is significantly influenced by gene locus or genomic location, nuclease platform, and cell type (summarized as a heat map in Fig. 6).

## Discussion

A central technological goal of genome-editing is the ability to efficiently generate high-fidelity, precise edits while minimizing generation of damaging insertions or deletions by NHEJ. We developed a rapid ddPCR–based strategy to simultaneously measure HDR and NHEJ events in genome-edited samples. This assay allows for systematic evaluation of a large number of genome-editing conditions and provides a quantitative readout of editing outcomes. Therefore, we evaluated various nuclease platforms at three gene loci in HEK293T cells, HeLa cells, and human iPSCs to find conditions that favor HDR over NHEJ. In our conditions, the nucleases we tested gave relatively low genome-editing frequencies (Figs 3, 4, 5). This may be because the gene loci we tested were difficult to edit and/or our transfection efficiency was not high compared to other reports. However, our ddPCR-based assay was so sensitive that we were able to elucidate the overall trends of the genome-editing outcomes with multiple different nucleases. In general, Cas9 had higher activity, especially to induce NHEJ, than Cas9 nickases, and FokI-dCas9 induced predominantly NHEJ with little HDR (Figs 3, 4, 5, 6 and Supplementary Figs S7–S10). However, the genome-editing outcomes were highly context-dependent (Fig. 6). For example, *RBM20* TALENs induced more HDR than NHEJ in human iPSCs but more NHEJ than HDR in HEK293T cells and HeLa cells. Moreover, although some of the Cas9 systems induced more HDR than NHEJ in HEK293T cells, none of them efficiently induced HDR at *RBM20* in human iPSCs or HeLa cells (Figs 3a,b and 4). In HeLa cells, induction of HDR was generally inefficient (Fig. 4a,b, and Supplementary Fig. 9). This cell-type dependency may reflect differential expression of components of the DNA repair machinery or different epigenetic modifications (e.g., chromatin state) in the three cell types. Genome-editing tools have relatively frequent off-target effects in commonly used cell lines, but very few in isolated human iPSCs^12^,^13^,^30^,^31^,^32^,^33^,^34^,^35^. These observations may also reflect a difference in active DNA repair mechanisms or different transfection efficacy in different cell types. It will be interesting to test more cell types and examine the expression of DNA repair components and their epigenetic status in those cell types.

In HEK293T cells, Cas9 nickases induced more HDR than NHEJ under some conditions (Figs 3 and 6 and Supplementary Fig. S7). In some cases, tandem nicking, especially by Cas9-H840A in *RBM20*, efficiently induced HDR with minimal NHEJ (Fig. 3b). To our knowledge, tandem nicking is a novel genome-editing strategy. We also found that with each combination and orientation of gRNAs, Cas9-D10A and Cas9-H840A had distinct activities. We hypothesize that these differences in the HDR/NHEJ ratio reflect differences in how the RuvC and HNH domains cleave DNA and/or in the accessibility of the DNA to the repair machinery^8^,^9^. As judged from biochemical studies, Cas9 remains bound to the cut sites even after it cleaves DNA^36^; the presence of Cas9 and the binding of gRNA to the complementary strand should affect the accessibility of the donor DNA to genomic DNA during activation of the HDR pathway as shown by recent biochemical study^10^. Moreover, Cas9-H840A but not Cas9-D10A has 3′ to 5′ exonuclease activity, which creates some space that may affect the interactions of donor DNA, DNA repair machinery, and genomic DNA^1^. These differences could explain why Cas9-D10A and Cas9-H840A behave so differently. These strand-specific modes of nicking DNA could also explain why sense and antisense strand donors give different editing outcomes. DSBs and DNA nicks are repaired by different pathways^37^,^38^. It would be interesting to study the DNA repair mechanisms by which different combinations of strand-specific nicks preferentially induce HDR. One possible explanation why antisense donors worked better than sense donors with in HEK293T cells when using Cas9-D10A and F1 gRNA for the RBM20 and GRN loci is that theoretically the induced nicks can be repaired by donor oligonucleotide assimilation^34^. Although the differences in Cas9-D10A and Cas9-H840A are quite clear from our experiments, the disparate results obtained at different gene loci and in different cell types will require further experimentation to elucidate the mechanisms involved.

NHEJ-inducing activity was generally higher in the presence of homologous donor oligonucleotides than in the absence of them (Supplementary Figs S7 and S8). It is possible that Cas9-gRNA complexes recognized the donor oligonucleotides complementary to gRNAs, and the interaction of the genomic DNA and the Cas9-gRNA complexes was affected. This hypothesis is consistent with the observation that sense and antisense donor oligonucleotides induced different levels of NHEJ with Cas9 (Supplementary Fig. 7).

We found no robust correlation between the frequencies of HDR and NHEJ induced by genome-editing (Fig. 5). In some studies, nuclease platforms’ activities were measured only by NHEJ^4^,^6^,^7^,^29^. However, our findings indicate a difficulty of estimating HDR-inducing activity based solely on NHEJ-inducing activity. For example, even though Cas9 generally induces much more frequent NHEJ than Cas9 nickases, they induce HDR at a similar level. Also, FokI-dCas9 induces NHEJ with a very low level of HDR (Figs 3, 4, 5, 6). Our findings suggest that HDR and NHEJ must be separately assessed to evaluate nuclease activities. Our novel method offers an ideal strategy to achieve this goal.

We developed and reported the sib-selection-based technique to isolate human pluripotent stem cells with point mutations^20^. However, NHEJ alleles could not be detected until clones were isolated using the original probe design, which can result in clones with NHEJ events (Supplementary Table S1). Therefore, we suggest that our simultaneous HDR and NHEJ detection assay to be used to optimize any genome-editing project where NHEJ is undesirable. With this assay, HDR and NHEJ alleles can be simultaneously detected to avoid clones with NHEJ events. Moreover, by testing multiple genome-editing conditions with our assay, the best conditions for inducing HDR can be identified for mutagenesis. If NHEJ is desired, the NHEJ assay can also be used to isolate cell lines with NHEJ events that disrupt gene functions. The frequency of NHEJ can be monitored with our assay and enrich for it by sib-selection. We have isolated iPSC lines with NHEJ alleles (Supplementary Fig. S12). Thus, our HDR and NHEJ detection system is a powerful tool to isolate cell clones.

Although our new assay system is powerful, improvements will be needed to keep pace with this rapidly moving field. For instance, genome-editing with larger donor DNA would be challenging for this ddPCR assay, because of the limitation of the size of PCR amplicons. In addition, as the costs of DNA sequencing continues to decline it will be more widely used to detect editing events that cannot be predicted. Given the rapid pace of development in DNA sequencing, ddPCR technology, and genome-editing, this is likely to remain a dynamic area of innovation. Currently the ddPCR method we describe is the most robust, rapid, and cost effective method that we have found for assessment of genome-editing outcomes.

For precise genome-editing, it is essential to have HDR with minimal NHEJ. Our method simultaneously assesses HDR and NHEJ with unprecedented sensitivity and reproducibility, without requiring isolation and sequencing of cell clones. Conditions favoring HDR over NHEJ differed at different loci, suggesting an unappreciated genomic topography that could involve epigenetic modification and DNA repair. Our method will be useful to deepen our understanding of DNA repair mechanisms induced by sequence-specific nucleases and may lead to more efficient and precise genome-editing protocols.

## Additional Information

**How to cite this article**: Miyaoka, Y. *et al*. Systematic quantification of HDR and NHEJ reveals effects of locus, nuclease, and cell type on genome -editing. *Sci. Rep.* **6**, 23549; doi: 10.1038/srep23549 (2016).

## Supplementary Material

Supplementary Information
