# Supplementary material for: Systematic quantification of HDR and NHEJ reveals effects of locus, nuclease, and cell type on genome-editing
Source: Sci Rep. 2016 Mar 31;6:23549. doi: 10.1038/srep23549 (PMC4814844; doi:10.1038/srep23549)
Supplement: Supplementary Information [file srep23549-s1.pdf]

Supplementary Information

Systematic quantification of HDR and NHEJ reveals effects of locus, nuclease, and cell type on genome editing

Yuichiro Miyaoka[1,†¶*], Jennifer R. Berman[2,†], Samantha B. Cooper[2], Steven J. Mayerl[1], Amanda H. Chan[1], Bin Zhang[2], George A. Karlin-Neumann[2], and Bruce R. Conklin[1,3,*]

[1] Gladstone Institute of Cardiovascular Disease, San Francisco, California, 94158, USA
[2] Digital Biology Center, Bio-Rad Laboratories, Pleasanton, California, 94588, USA
[3] Departments of Medicine and Cellular and Molecular Pharmacology, University of California, San Francisco, San Francisco, California, 94143, USA

* To whom correspondence should be addressed. Tel: +81 3 5316 3227; Fax: +81 3 5316 3150; Email: miyaoka-yi@igakuken.or.jp to Y.M. Tel: +1 415 734 2712; Fax: +1 415 355 0960; Email: bconklin@gladstone.ucsf.edu to B.R.C.

† These authors contributed equally to this work.

¶ Current affiliation: Regenerative Medicine Project, Tokyo Metropolitan Institute of Medical Science, Tokyo, 156-8506, Japan

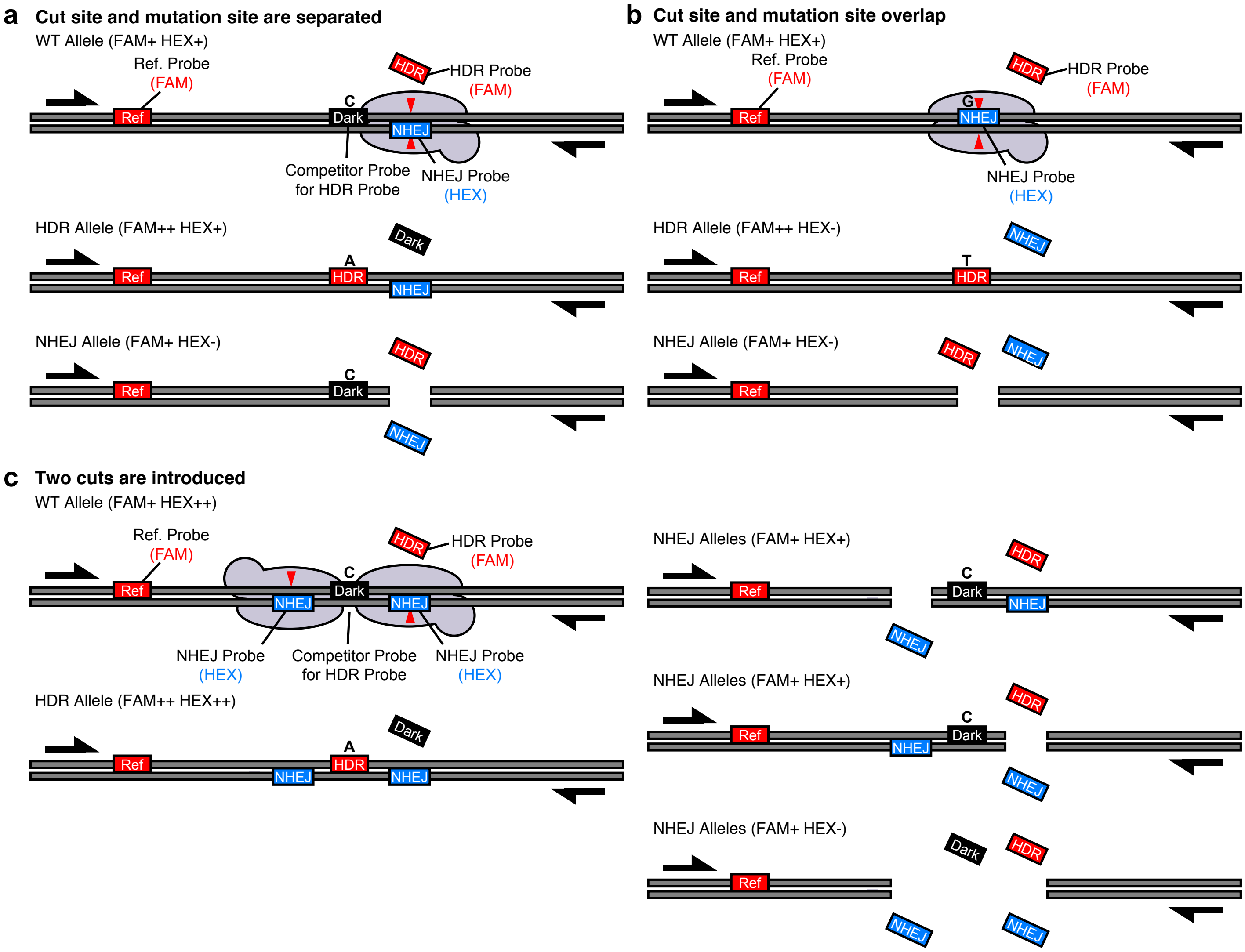
a
Cut site and mutation site are separated
WT Allele (FAM+ HEX+)
Ref. Probe
(FAM)
HDR Probe
(FAM)
Competitor Probe
for HDR Probe
NHEJ Probe
(HEX)
HDR Allele (FAM++ HEX+)
NHEJ Allele (FAM+ HEX-)
b
Cut site and mutation site overlap
WT Allele (FAM+ HEX+)
Ref. Probe
(FAM)
HDR Probe
(FAM)
NHEJ Probe
(HEX)
HDR Allele (FAM++ HEX-)
NHEJ Allele (FAM+ HEX-)
c
Two cuts are introduced
WT Allele (FAM+ HEX++)
Ref. Probe
(FAM)
HDR Probe
(FAM)
NHEJ Probe
(HEX)
Competitor Probe
for HDR Probe
NHEJ Probe
(HEX)
HDR Allele (FAM++ HEX++)
NHEJ Alleles (FAM+ HEX+)
NHEJ Alleles (FAM+ HEX+)
NHEJ Alleles (FAM+ HEX-)

Supplementary Figure S1. Varying the assay strategy by changing the number and position of cut sites. Depending on the editing strategy and the relative positions of cut site and edit site, the positions of assay probes and the need for a competitive blocking probe vary. (a) Assay design when cut site and mutation site are separated. The *RBM20* Cas9 with F1 gRNA assay is shown as an example (Supplementary Fig. S3). The HDR and NHEJ probes are located on nonoverlapping mutation and cut sites, respectively. Because these probes bind to different locations, a dark, nonextendible probe that competes with the HDR probe for WT allele binding is included. (b) Assay design when cut site and mutation site overlap. In these cases, the NHEJ probe overlaps with and is on the same strand as the HDR probe. The NHEJ probe thus competes with the HDR probe for WT binding and a dark probe is not necessary. Also, the HDR allele becomes FAM++ and HEX-. The *ATP7B* Cas9 with F1 gRNA assay is shown as an example (Supplementary Fig. S3). (c) Assay design when two cut sites are introduced. The *RBM20* dual Cas9-D10A with F1 and R1 gRNA assay is shown as an example (Supplementary Fig. S3). Because dual Cas9 systems introduce two cuts, two NHEJ probes are included in the assay. When neither NHEJ probe competes with an HDR probe, a dark probe is also designed. Because two NHEJ probes are included in this assay, the WT allele is detected as FAM+ and HEX++, and the NHEJ alleles are detected as FAM+ and HEX+, or FAM+ and HEX-. The reference and HDR probes are constant regardless of the location or number of cut sites, and at least one of the two primers is outside the donor DNA sequence to ensure quantification of integrated events.

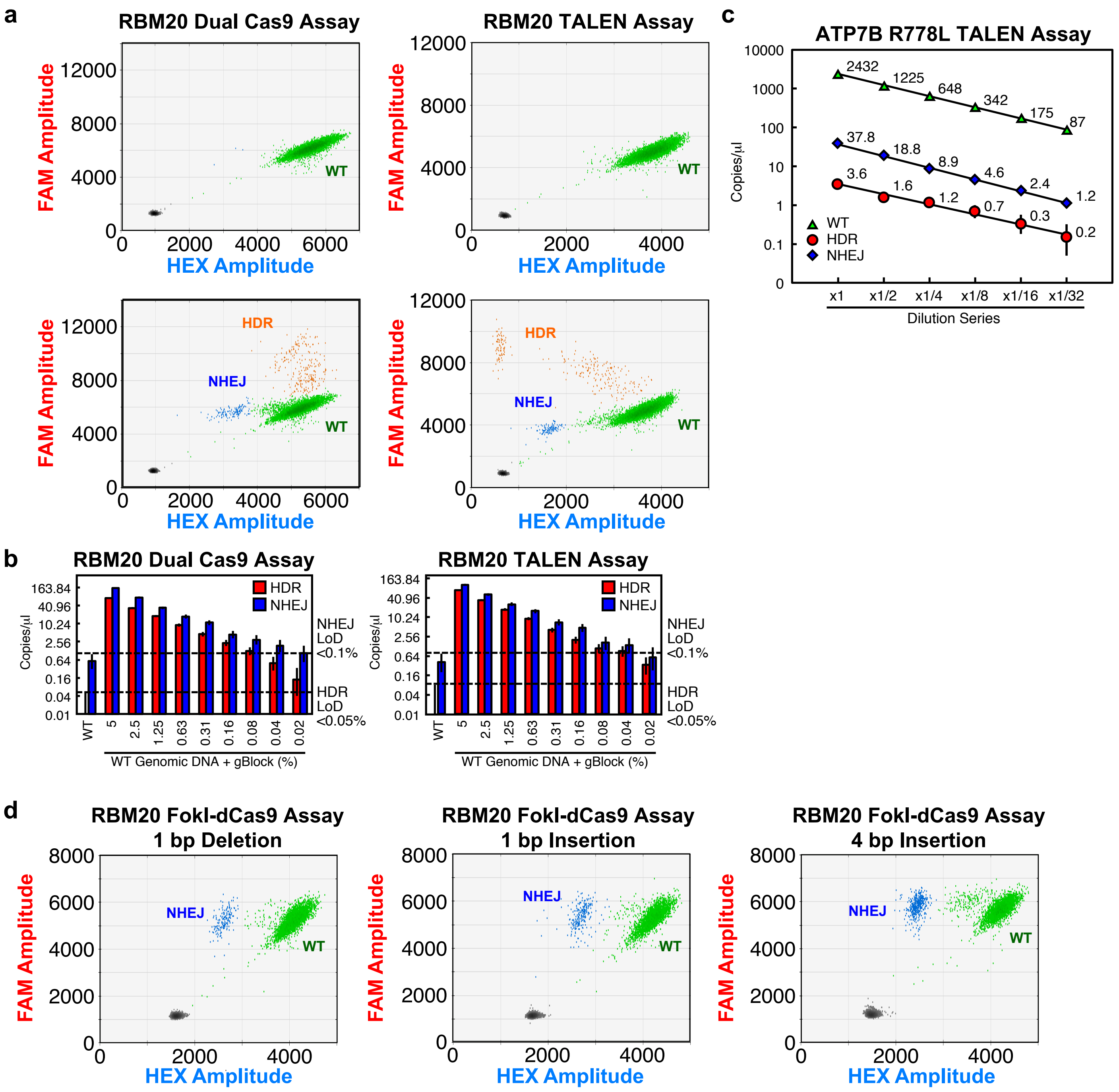
a
RBM20 Dual Cas9 Assay
FAM Amplitude
HEX Amplitude
0
2000
4000
6000
8000
12000
WT
RBM20 TALEN Assay
FAM Amplitude
HEX Amplitude
0
2000
4000
8000
12000
WT
HDR
NHEJ
WT
HDR
NHEJ
WT
b
RBM20 Dual Cas9 Assay
Copies/µl
163.84
40.96
10.24
2.56
0.64
0.16
0.04
0.01
HDR
NHEJ
NHEJ LoD <0.1%
HDR LoD <0.05%
WT
5
2.5
1.25
0.63
0.31
0.16
0.08
0.04
0.02
WT Genomic DNA + gBlock (%)
RBM20 TALEN Assay
Copies/µl
HDR
NHEJ
NHEJ LoD <0.1%
HDR LoD <0.05%
WT Genomic DNA + gBlock (%)
c
ATP7B R778L TALEN Assay
Copies/µl
10000
1000
100
10
1
0.1
0
2432
1225
648
342
175
87
37.8
18.8
8.9
4.6
2.4
1.2
3.6
1.6
1.2
0.7
0.3
0.2
WT
HDR
NHEJ
x1
x1/2
x1/4
x1/8
x1/16
x1/32
Dilution Series
d
RBM20 FokI-dCas9 Assay
1 bp Deletion
RBM20 FokI-dCas9 Assay
1 bp Insertion
RBM20 FokI-dCas9 Assay
4 bp Insertion
FAM Amplitude
HEX Amplitude
0
2000
4000
6000
8000
NHEJ
WT

Supplementary Figure S2. Using synthetic DNA to validate the assay to detect HDR and NHEJ. ddPCR assays to detect HDR and NHEJ edits simultaneously were validated on WT genomic DNA combined with synthetic double-stranded DNA (gBlocks, Supplementary Table S7) representing HDR-edited alleles and NHEJ-edited alleles (see Methods). (a) Representative two-dimensional droplet fluorescence amplitude plots of the *RBM20* dual Cas9 and TALEN assays on a WT genomic DNA-only well (upper plots, 100 ng input each) and a well containing 1% (lower wells, ~300 copies/well) of each mutant HDR or NHEJ gBlock. Droplets containing NHEJ (blue), HDR (orange), and WT (green) template are labeled. Fluorescence amplitude of droplet clusters can vary by assay strategy (Supplementary Fig. S1). In the TALEN assay, the HDR allele was detected as FAM++ and HEX- when present in a single positive droplet because the HDR and NHEJ probes bind mutually exclusively to the same region (Supplementary Fig. S1). (b) Assay sensitivity was established by twofold serial dilution of HDR and NHEJ synthetic template in a constant (100 ng) background of WT genomic DNA. The limit of detection (LoD) was ~0.1% for NHEJ and <0.05% for HDR, as established by comparison with WT genomic DNA-only wells (nonoverlap of 95% confidence intervals, dotted line). Data represent two merged wells per dilution point, and four merged wells for WT genomic DNA-only negative control. The 95% confidence interval is shown. (c) Linearity of HDR, NHEJ, and WT allele measurement over a twofold dilution series on a representative *ATP7B* TALEN-edited genomic DNA isolated from HEK293T cells. Dilution from 150 ng to 5 ng sample input is shown. (d) Detection of NHEJ alleles with insertions and deletions. Use of the *RBM20* FokI-dCas9 assay on synthetic template representing NHEJ insertion mutations (1 basepair and 4 basepair, Supplementary Table S7) showed comparable cluster positions to use on a synthetic template with a 1-basepair deletion. Accurate quantification of the deletion and insertion gBlocks was confirmed by an orthogonal assay specific to the gBlock (not shown).

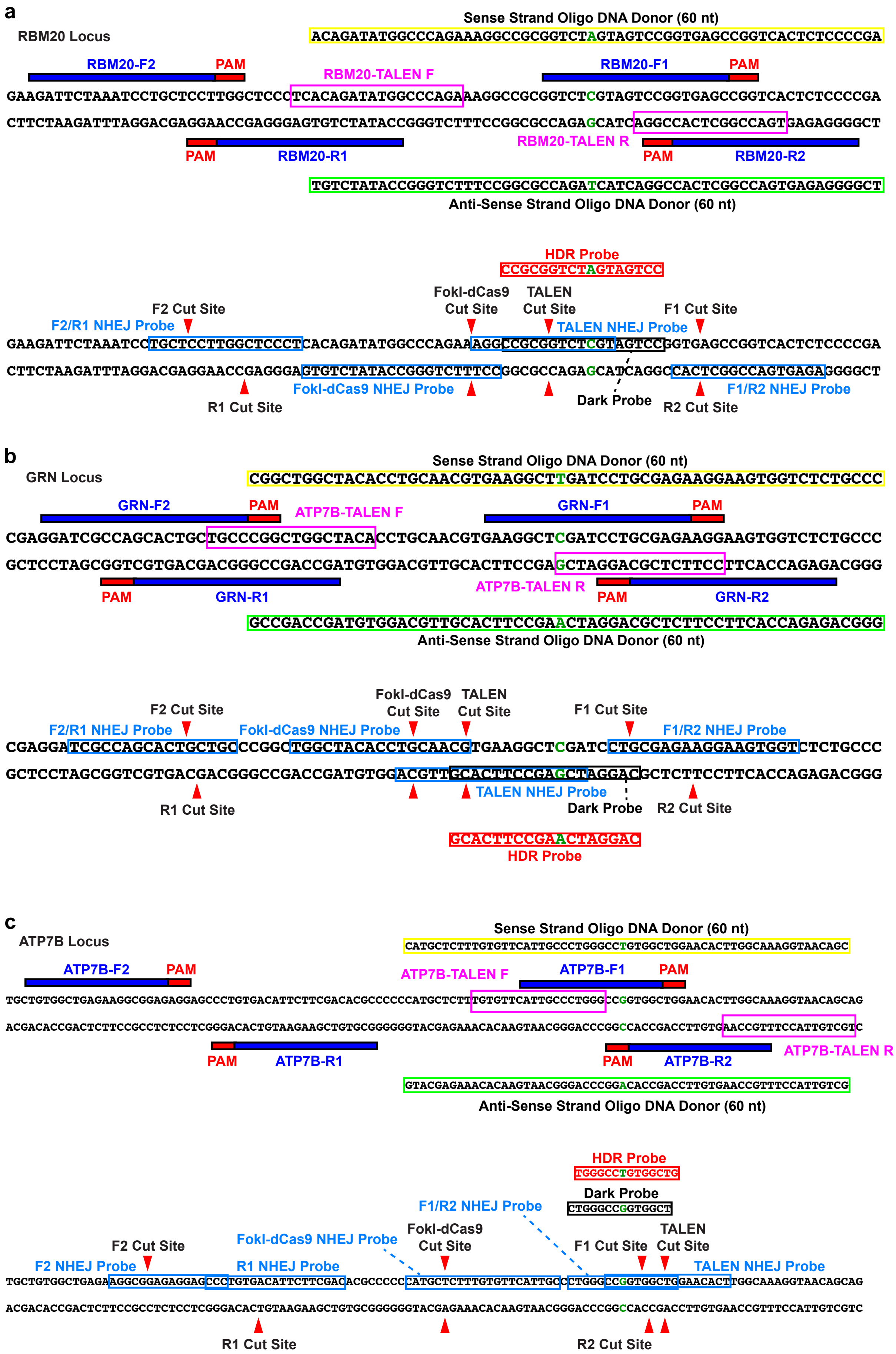
a
RBM20 Locus
Sense Strand Oligo DNA Donor (60 nt)
ACAGATATGGCCCAGAAAGGCCGCGGTCTAGTAGTCCGGTGAGCCGGTCACTCTCCCCGA
RBM20-F2
PAM
RBM20-TALEN F
RBM20-F1
PAM
GAAGATTCTAAATCCTGCTCCTTGGCTCCCTCACAGATATGGCCCAGAAAGGCCGCGGTCTCGTAGTCCGGTGAGCCGGTCACTCTCCCCGA
CTTCTAAGATTTAGGACGAGGAACCGAGGGAGTGTCTATACCGGGTCTTTCCGGCGCCAGAGCATCAGGCCACTCGGCCAGTGAGAGGGGCT
PAM
RBM20-R1
RBM20-TALEN R
PAM
RBM20-R2
TGTCTATACCGGGTCTTTCCGGCGCCAGATCATCAGGCCACTCGGCCAGTGAGAGGGGCT
Anti-Sense Strand Oligo DNA Donor (60 nt)
HDR Probe
CCGCGGTCTAGTAGTCC
FokI-dCas9 Cut Site
TALEN Cut Site
F2 Cut Site
F1 Cut Site
F2/R1 NHEJ Probe
TALEN NHEJ Probe
GAAGATTCTAAATCCTGCTCCTTGGCTCCCTCACAGATATGGCCCAGAAAGGCCGCGGTCTCGTAGTCCGGTGAGCCGGTCACTCTCCCCGA
CTTCTAAGATTTAGGACGAGGAACCGAGGGAGTGTCTATACCGGGTCTTTCCGGCGCCAGAGCATCAGGCCACTCGGCCAGTGAGAGGGGCT
FokI-dCas9 NHEJ Probe
F1/R2 NHEJ Probe
Dark Probe
R1 Cut Site
R2 Cut Site
b
GRN Locus
Sense Strand Oligo DNA Donor (60 nt)
CGGCTGGCTACACCTGCAACGTGAAGGCTTGATCCTGCGAGAAGGAAGTGGTCTCTGCCC
GRN-F2
PAM
ATP7B-TALEN F
GRN-F1
PAM
CGAGGATCGCCAGCACTGCTGCCCGGCTGGCTACACCTGCAACGTGAAGGCTCGATCCTGCGAGAAGGAAGTGGTCTCTGCCC
GCTCCTAGCGGTCGTGACGACGGGCCGACCGATGTGGACGTTGCACTTCCGAGCTAGGACGCTCTTCCTTCACCAGAGACGGG
PAM
GRN-R1
ATP7B-TALEN R
PAM
GRN-R2
GCCGACCGATGTGGACGTTGCACTTCCGAACTAGGACGCTCTTCCTTCACCAGAGACGGG
Anti-Sense Strand Oligo DNA Donor (60 nt)
FokI-dCas9 Cut Site
TALEN Cut Site
F2 Cut Site
F1 Cut Site
F2/R1 NHEJ Probe
FokI-dCas9 NHEJ Probe
F1/R2 NHEJ Probe
CGAGGATCGCCAGCACTGCTGCCCGGCTGGCTACACCTGCAACGTGAAGGCTCGATCCTGCGAGAAGGAAGTGGTCTCTGCCC
GCTCCTAGCGGTCGTGACGACGGGCCGACCGATGTGGACGTTGCACTTCCGAGCTAGGACGCTCTTCCTTCACCAGAGACGGG
TALEN NHEJ Probe
Dark Probe
R1 Cut Site
R2 Cut Site
GCACTTCCGAACTAGGAC
HDR Probe
c
ATP7B Locus
Sense Strand Oligo DNA Donor (60 nt)
CATGCTCTTTGTGTTCATTGCCCTGGGCCTGTGGCTGGAACACTTGGCAAAGGTAACAGC
ATP7B-F2
PAM
ATP7B-TALEN F
ATP7B-F1
PAM
TGCTGTGGCTGAGAAGGCGGAGAGGAGCCCTGTGACATTCTTCGACACGCCCCCCATGCTCTTTGTGTTCATTGCCCTGGGCCGGTGGCTGGAACACTTGGCAAAGGTAACAGCAG
ACGACACCGACTCTTCCGCCTCTCCTCGGGACACTGTAAGAAGCTGTGCGGGGGGTACGAGAAACACAAGTAACGGGACCCGGCCACCGACCTTGTGAACCGTTTCCATTGTCGTC
PAM
ATP7B-R1
PAM
ATP7B-R2
ATP7B-TALEN R
GTACGAGAAACACAAGTAACGGGACCCGGACACCGACCTTGTGAACCGTTTCCATTGTCG
Anti-Sense Strand Oligo DNA Donor (60 nt)
HDR Probe
TGGGCCTGTGGCTG
Dark Probe
CTGGGCCGGTGGCT
F1/R2 NHEJ Probe
FokI-dCas9 NHEJ Probe
FokI-dCas9 Cut Site
TALEN Cut Site
F2 Cut Site
F1 Cut Site
F2 NHEJ Probe
R1 NHEJ Probe
TALEN NHEJ Probe
TGCTGTGGCTGAGAAGGCGGAGAGGAGCCCTGTGACATTCTTCGACACGCCCCCCATGCTCTTTGTGTTCATTGCCCTGGGCCGGTGGCTGGAACACTTGGCAAAGGTAACAGCAG
ACGACACCGACTCTTCCGCCTCTCCTCGGGACACTGTAAGAAGCTGTGCGGGGGGTACGAGAAACACAAGTAACGGGACCCGGCCACCGACCTTGTGAACCGTTTCCATTGTCGTC
R1 Cut Site
R2 Cut Site

Supplementary Figure S3. Assay design to detect HDR and NHEJ. (a–c) Assay design for *RBM20* (a), *GRN* (b), and *ATP7B* (c). The locations of gRNAs, TALENs, and donor DNAs are shown in the top panels. The mutation sites are highlighted in green. The locations of HDR, NHEJ, and dark probes are shown in the bottom panels. The locations of probes for individual *RBM20* assays are also shown in Fig. 2 without the dark probe. For simplicity, primers and reference probes are not included here (see Supplementary Table S5 for their sequences). In *ATP7B*, because the predicted cut sites of F2 and R1 gRNAs are not close enough, two NHEJ probes were designed for them. In *RBM20* and *GRN*, but not *ATP7B*, the single F2/R1 NHEJ probes cover both F2 and R1 gRNA cut sites. For some assays (those including *RBM20*, *GRN*, and *ATP7B* TALEN probes or *ATP7B* F1/R2 probe; Supplementary Table S6), the dark probe is omitted since the NHEJ probe directly overlaps the HDR probe and serves as a competitive binder for the WT sequence (Supplementary Fig. S1). Red triangles indicate the predicted cut sites by nucleases. HDR probes specifically bind to alleles induced by HDR, whereas NHEJ probes lose their binding sites when insertions or deletions are created by NHEJ.

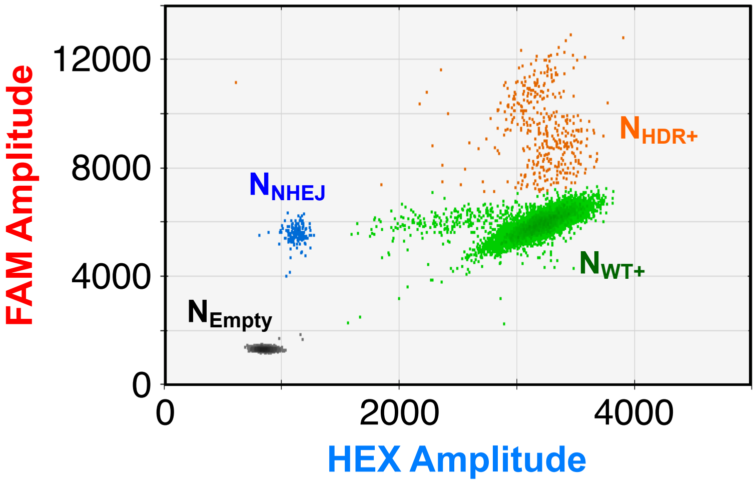

FAM Amplitude
12000
8000
4000
0
0
2000
4000
HEX Amplitude
N_{HDR+}
N_{NHEJ}
N_{WT+}
N_{Empty}

Supplementary Figure S4. Droplet group definitions for analysis. In two-dimensional plots, droplets without any templates were gated as the black population (N_{Empty}), while all droplets positive for HDR (FAM++) were gated as an orange population (N_{HDR+}). Droplets containing only NHEJ alleles (FAM+ HEX-) were gated as a blue population (N_{NHEJ}). All other droplets were gated as a green population (N_{WT+}). These definitions were used to calculate the HDR and NHEJ allelic frequencies (see Methods).

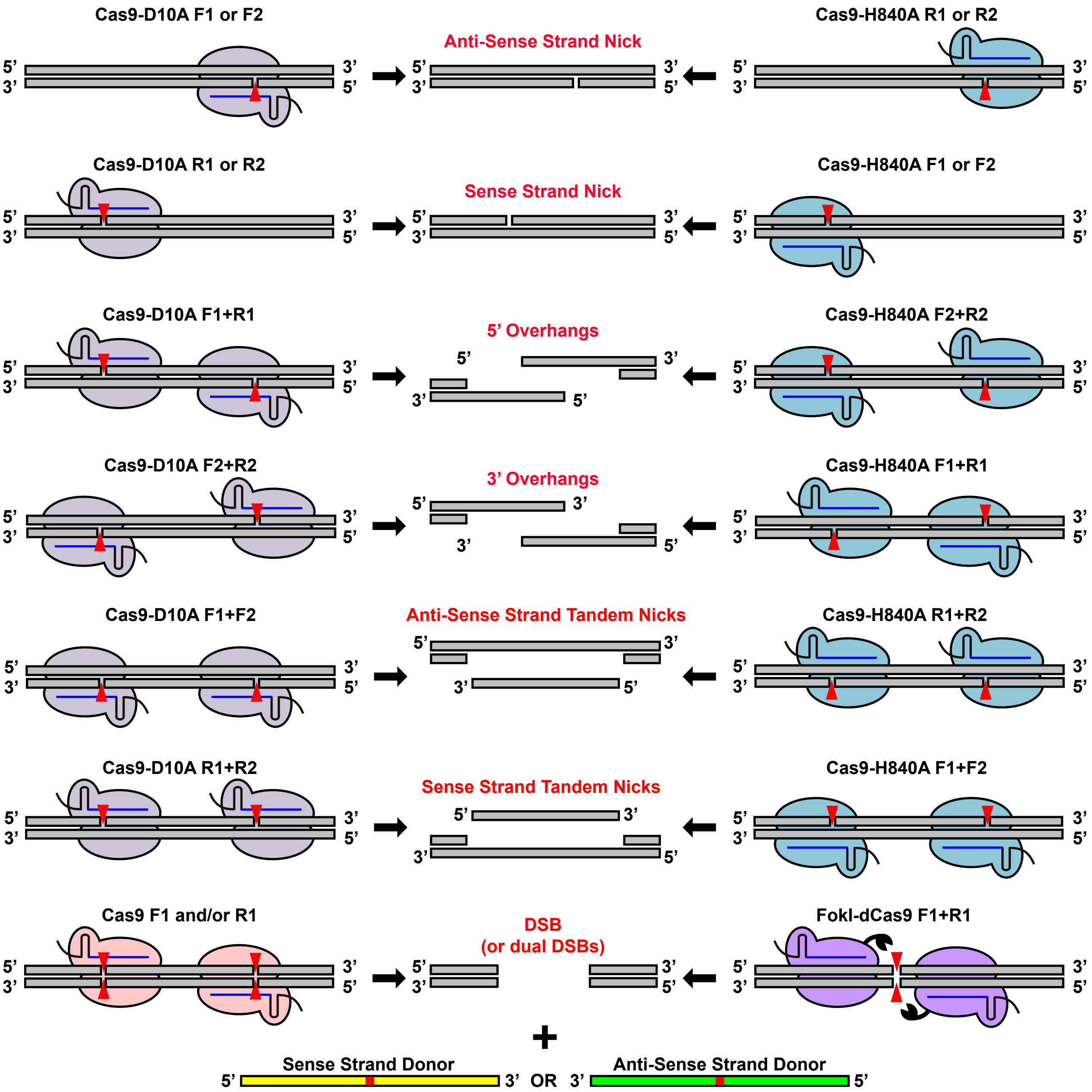
Cas9-D10A F1 or F2
Anti-Sense Strand Nick
Cas9-H840A R1 or R2
Cas9-D10A R1 or R2
Sense Strand Nick
Cas9-H840A F1 or F2
Cas9-D10A F1+R1
5' Overhangs
Cas9-H840A F2+R2
Cas9-D10A F2+R2
3' Overhangs
Cas9-H840A F1+R1
Cas9-D10A F1+F2
Anti-Sense Strand Tandem Nicks
Cas9-H840A R1+R2
Cas9-D10A R1+R2
Sense Strand Tandem Nicks
Cas9-H840A F1+F2
Cas9 F1 and/or R1
DSB
(or dual DSBs)
FokI-dCas9 F1+R1
+
Sense Strand Donor
OR
Anti-Sense Strand Donor
5'
3'

Supplementary Figure S5. Schematic representation of genomic modifications by Cas9 platforms tested in this study. Different combinations of gRNAs and nuclease platforms generate a wide variety of conditions, including single-strand nicking, double-strand nicking, and tandem nicking. TALENs also induce DSBs (not shown).

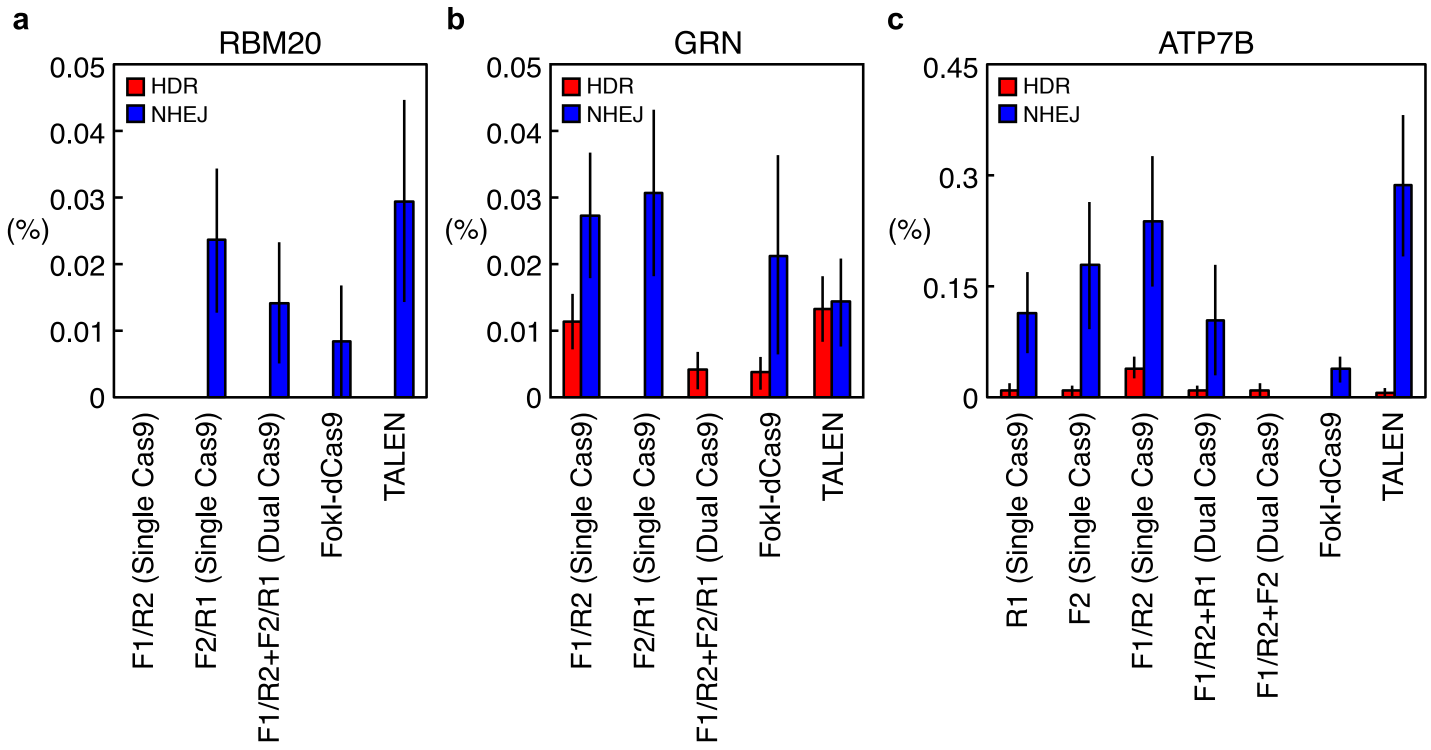
a
RBM20
b
GRN
c
ATP7B
HDR
NHEJ
(%)
0.05
0.04
0.03
0.02
0.01
0
0.45
0.3
0.15
F1/R2 (Single Cas9)
F2/R1 (Single Cas9)
F1/R2+F2/R1 (Dual Cas9)
FokI-dCas9
TALEN
R1 (Single Cas9)
F2 (Single Cas9)
F1/R2+R1 (Dual Cas9)
F1/R2+F2 (Dual Cas9)

Supplementary Figure S6. Background signal of the assay to detect HDR and NHEJ. (a–c) HDR and NHEJ signals obtained by analyses of control genomic DNA for *RBM20* (a), *GRN* (b), and *ATP7B* (c). HEK293T cells were treated with gene-specific oligonucleotide DNA donors and Cas9, but with gRNAs targeting unrelated genomic regions as negative controls. Genomic DNA isolated from these cells was analyzed with our assay system. For this test, similar sample inputs were used (100–150 ng of genomic DNA) as assessed with a spectrophotometer, although amplifiable copies varied from 2,000–40,000 copies per well. The *RBM20* assay showed no HDR background and minimal (<0.05%) NHEJ background. The *GRN* assay showed minimal (<0.05%) HDR and NHEJ backgrounds. The *ATP7B* assay showed minimal (<0.05%) HDR and relatively high (up to 0.3%) NHEJ background noise. These background values were subtracted from the results obtained from samples treated with gene-specific nucleases.

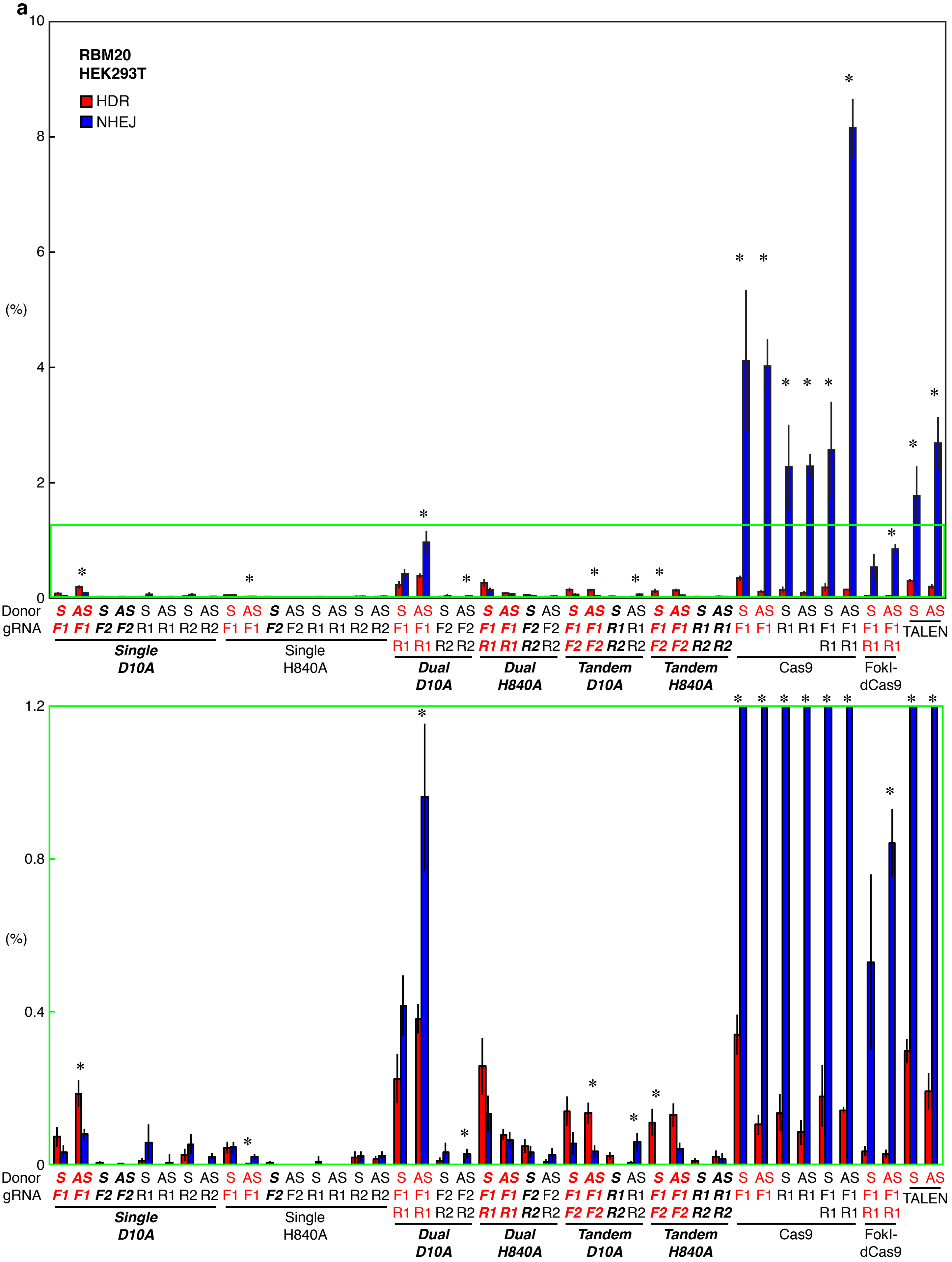
a
RBM20
HEK293T
HDR
NHEJ
(%)
10
8
6
4
2
0
1.2
0.8
0.4
Donor
gRNA
Single D10A
Single H840A
Dual D10A
Dual H840A
Tandem D10A
Tandem H840A
Cas9
FokI-dCas9
TALEN
S AS S AS S AS S AS
F1 F1 F2 F2 R1 R1 R2 R2

b

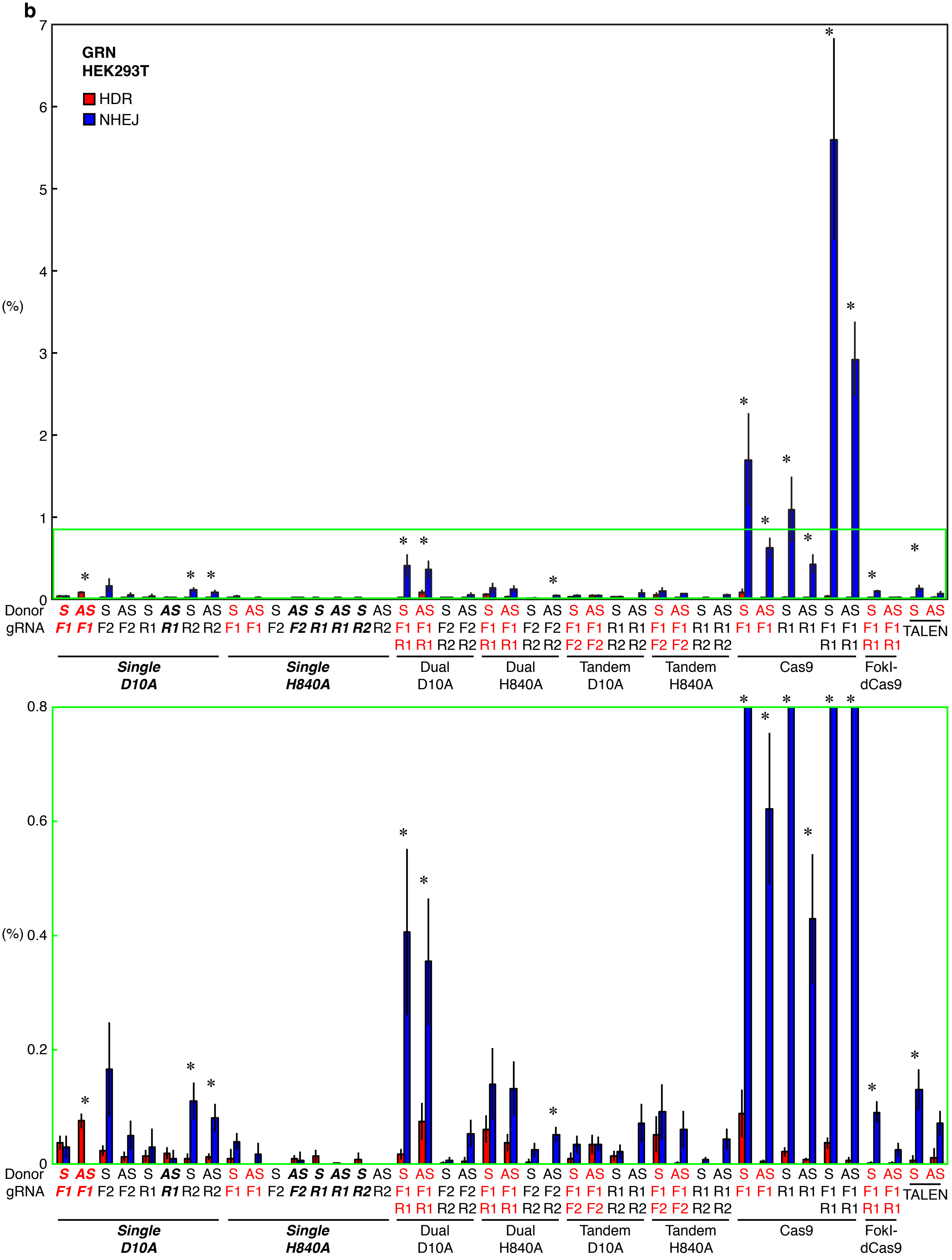
GRN
HEK293T
HDR
NHEJ
(%)
Donor
gRNA
S AS S AS S AS S AS S AS S AS S AS S AS S AS S AS S AS S AS S AS S AS S AS S AS S AS S AS S AS S AS S AS
F1 F1 F2 F2 R1 R1 R2 R2 F1 F1 F2 F2 R1 R1 R2 R2 F1 F1 F2 F2 F1 F1 F2 F2 F1 F1 R1 R1 F1 F1 R1 R1 F1 F1 R1 R1 F1 F1 F1 F1 TALEN
R1 R1 R2 R2 R1 R1 R2 R2 F2 F2 R2 R2 F2 F2 R2 R2 R1 R1 R1 R1
Single D10A
Single H840A
Dual D10A
Dual H840A
Tandem D10A
Tandem H840A
Cas9
FokI-dCas9

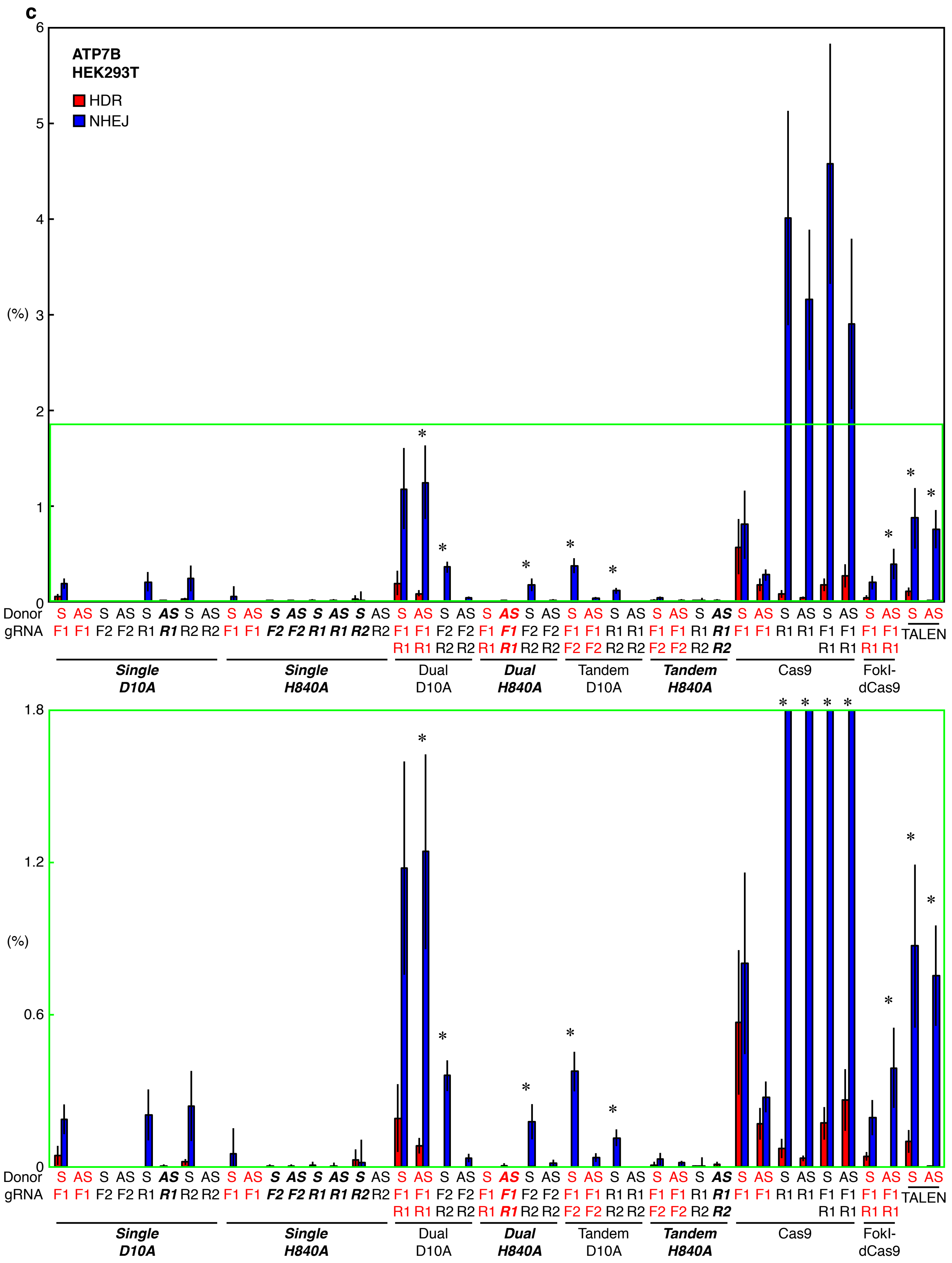
c
ATP7B
HEK293T
HDR
NHEJ
(%)
0
1
2
3
4
5
6
Donor
gRNA
S AS S AS S AS S AS S AS S AS S AS S AS S AS S AS S AS S AS S AS S AS S AS S AS S AS S AS S AS S AS S AS S AS S AS S AS
F1 F1 F2 F2 R1 R1 R2 R2 F1 F1 F2 F2 R1 R1 R2 R2 F1 F1 F2 F2 F1 F1 F2 F2 F1 F1 R1 R1 F1 F1 R1 R1 F1 F1 R1 R1 F1 F1 F1 F1 F1 F1
R1 R1 R2 R2 R1 R1 R2 R2 F2 F2 R2 R2 F2 F2 R2 R2 R1 R1 R1 R1
TALEN
Single D10A
Single H840A
Dual D10A
Dual H840A
Tandem D10A
Tandem H840A
Cas9
FokI-dCas9
(%)
0
0.6
1.2
1.8

Supplementary Figure S7. Complete sets of assay results of HDR and NHEJ induced by sequence-specific nucleases in HEK293T cells. (a–c) Genome-editing outcomes at *RBM20* (a), *GRN* (b), and *ATP7B* (c) in HEK293T cells. HDR (red) and NHEJ (blue) allelic frequency (%) is shown. The results are shown in two different scales (highlighted in green). Conditions that gave equivalent or more HDR than NHEJ are highlighted by bold italic characters. Also, conditions summarized as a heat map in Fig. 6 are highlighted by red characters. Values are mean ± SEM. n = 6. The differences between the HDR and NHEJ frequencies were evaluated by Student's T-test (*, $p<0.05$). S, sense strand oligonucleotide donor; AS, antisense strand oligonucleotide donor. The background signals of the assays were subtracted (Supplementary Fig. S6).

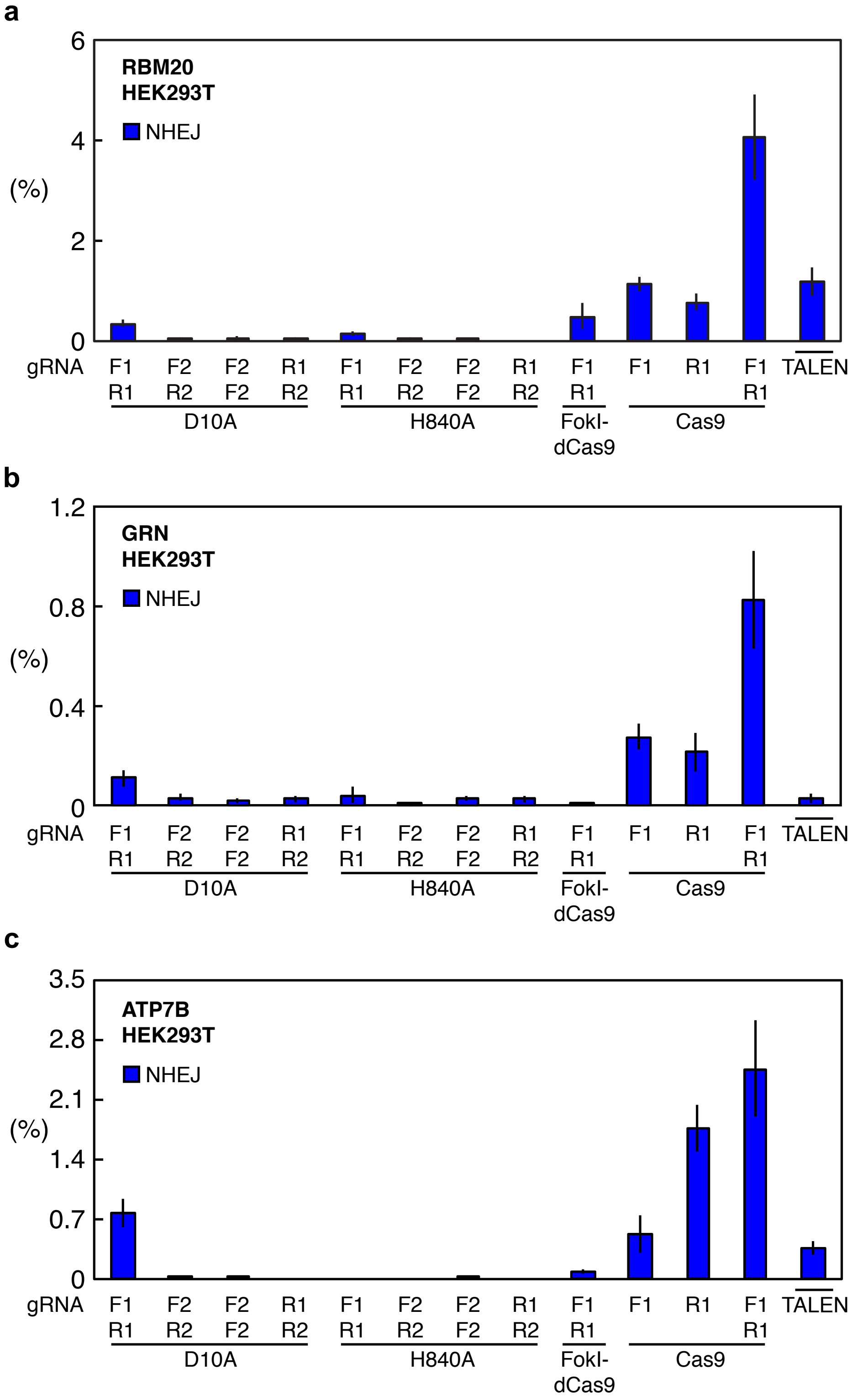
a
RBM20
HEK293T
NHEJ
(%)
6
4
2
0
gRNA
F1 R1
F2 R2
F2 F2
R1 R2
F1 R1
F2 R2
F2 F2
R1 R2
F1 R1
F1
R1
F1 R1
TALEN
D10A
H840A
FokI-dCas9
Cas9
b
GRN
HEK293T
NHEJ
(%)
1.2
0.8
0.4
0
gRNA
F1 R1
F2 R2
F2 F2
R1 R2
F1 R1
F2 R2
F2 F2
R1 R2
F1 R1
F1
R1
F1 R1
TALEN
D10A
H840A
FokI-dCas9
Cas9
c
ATP7B
HEK293T
NHEJ
(%)
3.5
2.8
2.1
1.4
0.7
0
gRNA
F1 R1
F2 R2
F2 F2
R1 R2
F1 R1
F2 R2
F2 F2
R1 R2
F1 R1
F1
R1
F1 R1
TALEN
D10A
H840A
FokI-dCas9
Cas9

Supplementary Figure S8. Measurement of NHEJ induced by sequence-specific nucleases in the absence of homologous donor DNA in HEK293T cells. (a–c) NHEJ-inducing activity at *RBM20* (a), *GRN* (b), and *ATP7B* (c) in HEK293T cells. NHEJ (blue) allelic frequency (%) is shown. As negative controls, the sense oligonucleotide donor DNA for GRN was used for *RBM20* and *ATP7B*, and the oligonucleotide donor DNA for *ATP7B* was used for *GRN*. The overall trend was the same as that in the presence of donor DNA in that Cas9 produced more NHEJ than Cas9 nickases, especially with both F1 and R1 gRNAs, but the activity was generally lower than in the presence of donor DNA. Values are mean ± SEM. n = 6. The background signals of assays from equivalent amounts of WT gDNA were subtracted (Supplementary Fig. S6). NHEJ-only measurements can be done with an assay lacking HDR and dark probes if desired.

a

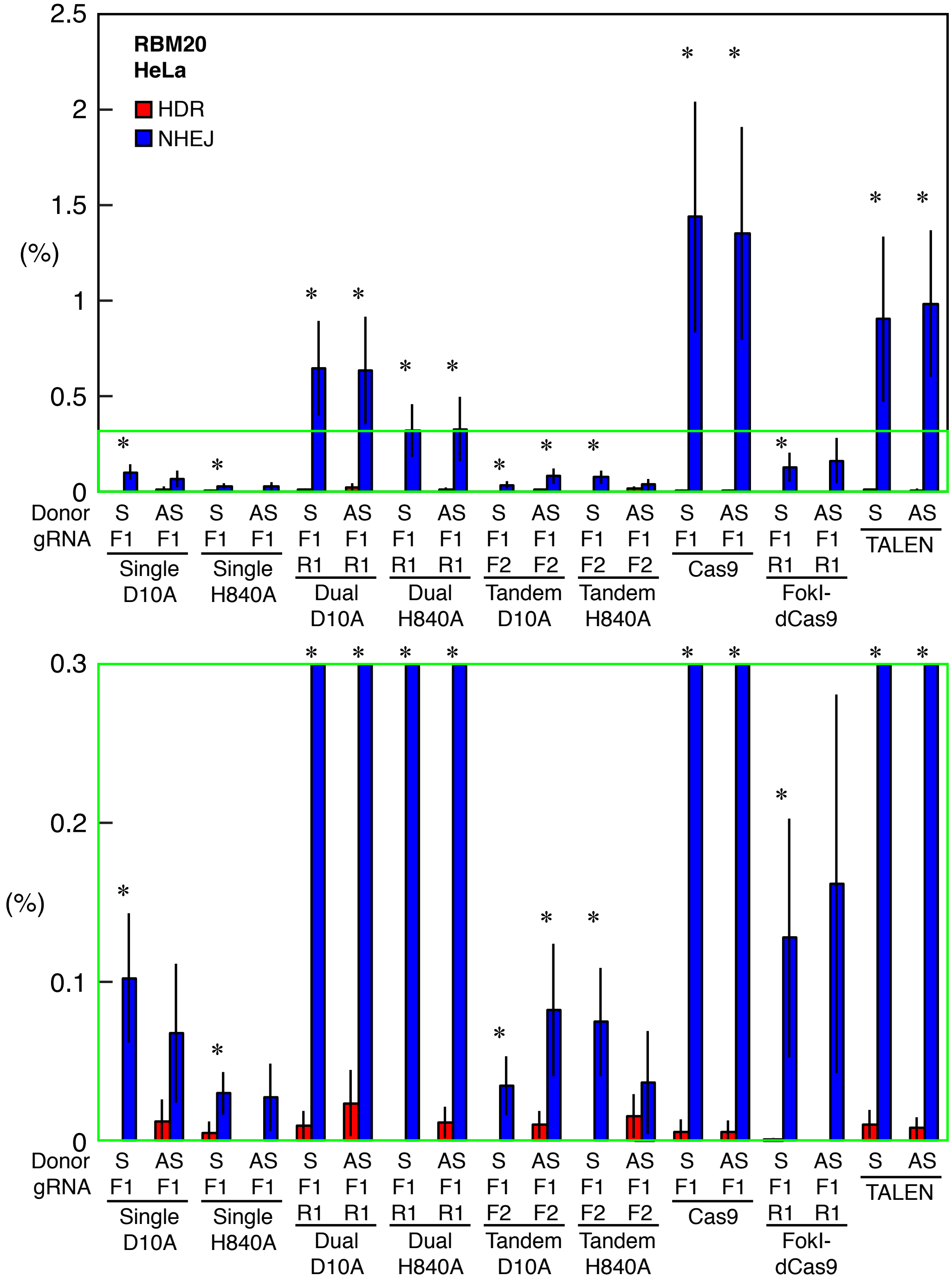

RBM20
HeLa
HDR
NHEJ
(%)
2.5
2
1.5
1
0.5
0
0.3
0.2
0.1
Donor
gRNA
S AS S AS S AS S AS S AS S AS S AS S AS S AS
F1 F1 F1 F1 F1 F1 F1 F1 F1 F1 F1 F1 F1 F1 F1 F1
R1 R1 R1 R1 F2 F2 F2 F2 R1 R1
Single D10A
Single H840A
Dual D10A
Dual H840A
Tandem D10A
Tandem H840A
Cas9
FokI-dCas9
TALEN

b

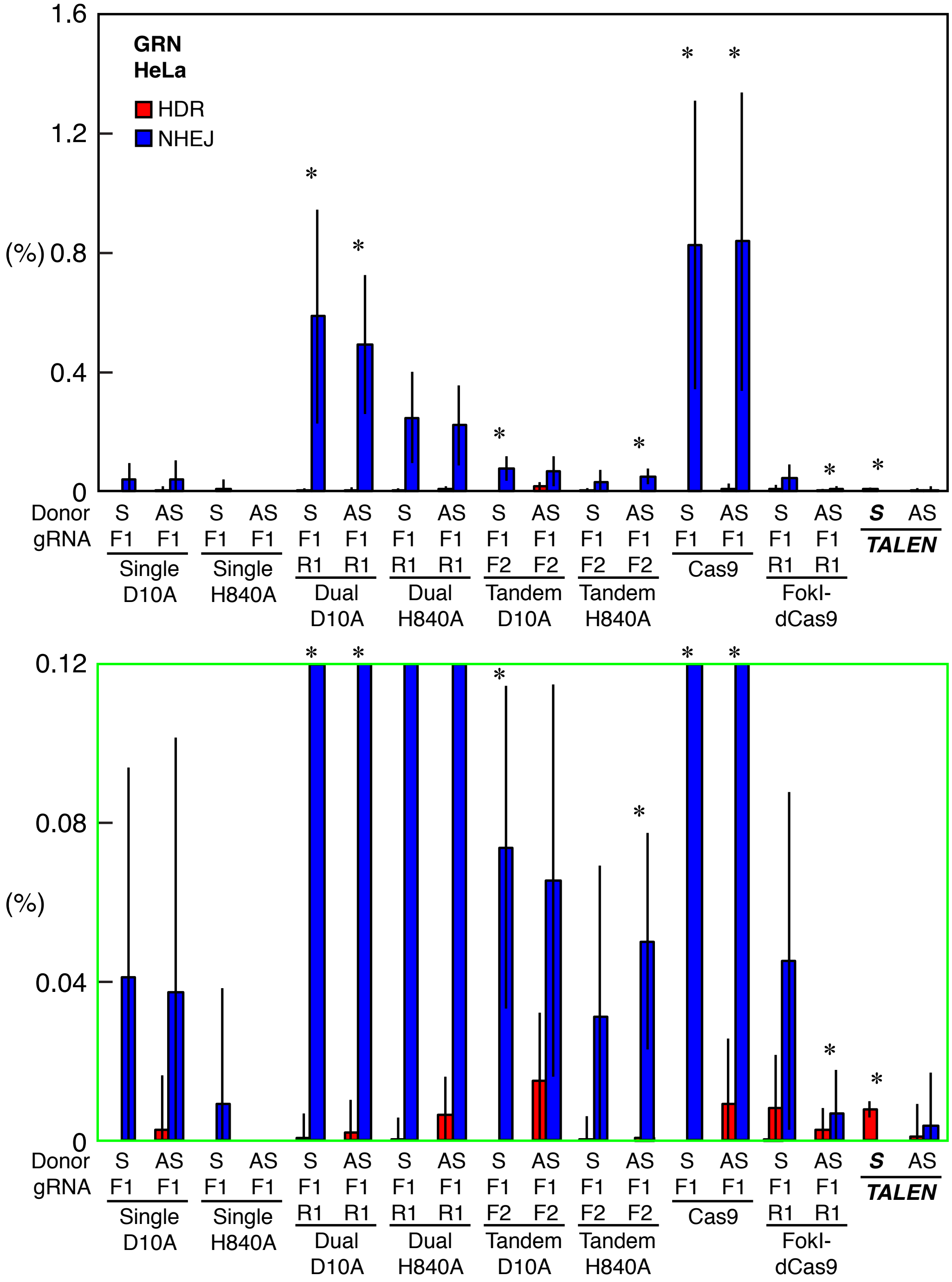

GRN
HeLa
HDR
NHEJ
1.6
1.2
(%) 0.8
0.4
0
Donor
gRNA
S F1 AS F1 Single D10A
S F1 AS F1 Single H840A
S F1 R1 AS F1 R1 Dual D10A
S F1 R1 AS F1 R1 Dual H840A
S F1 F2 AS F1 F2 Tandem D10A
S F1 F2 AS F1 F2 Tandem H840A
S F1 AS F1 Cas9
S F1 R1 AS F1 R1 FokI-dCas9
S AS TALEN
0.12
0.08
(%)
0.04
0
Donor
gRNA
S F1 AS F1 Single D10A
S F1 AS F1 Single H840A
S F1 R1 AS F1 R1 Dual D10A
S F1 R1 AS F1 R1 Dual H840A
S F1 F2 AS F1 F2 Tandem D10A
S F1 F2 AS F1 F2 Tandem H840A
S F1 AS F1 Cas9
S F1 R1 AS F1 R1 FokI-dCas9
S AS TALEN

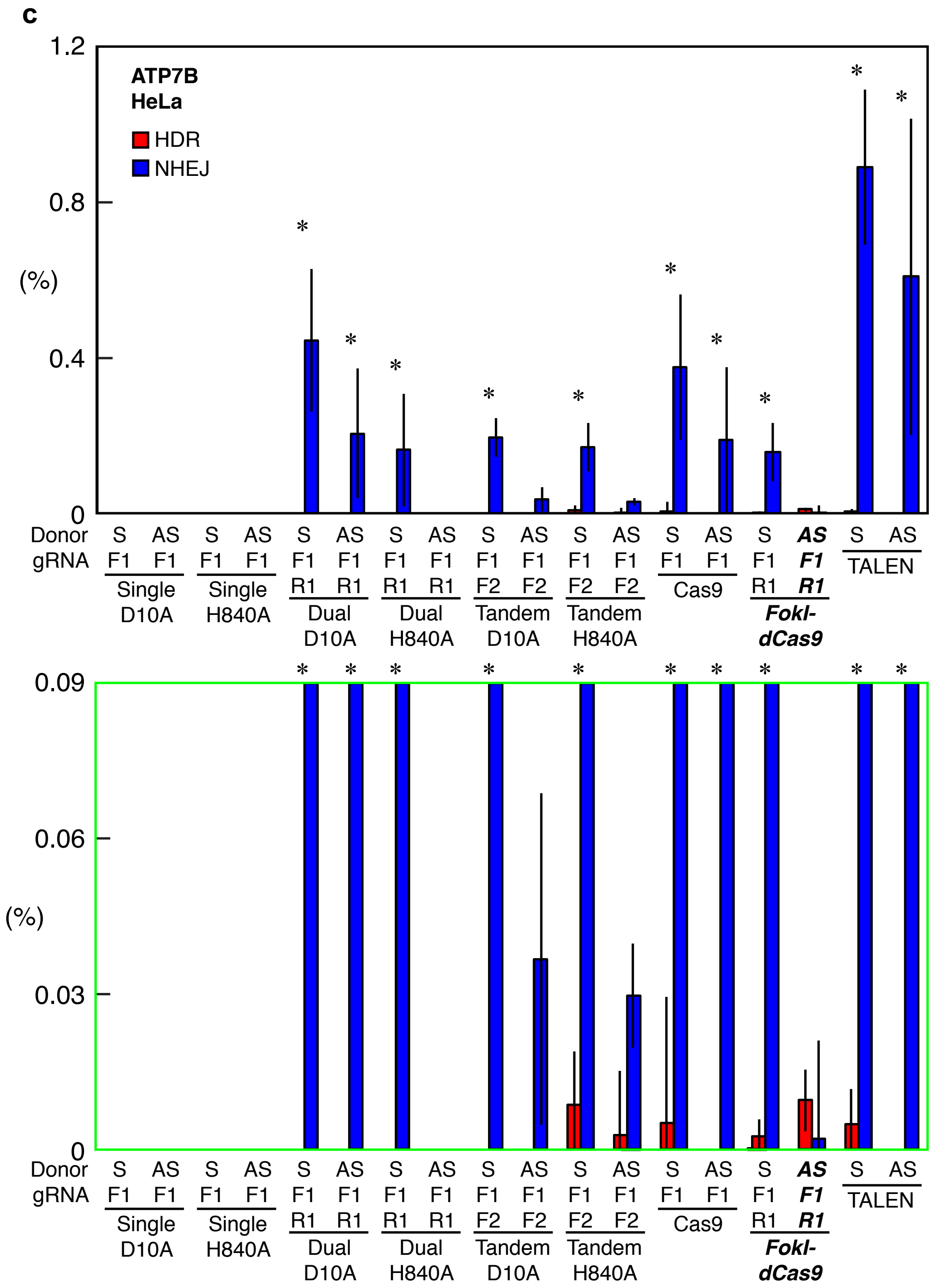
c
ATP7B
HeLa
HDR
NHEJ
1.2
0.8
0.4
0
(%)
0.09
0.06
0.03
Donor
gRNA
S AS S AS S AS S AS S AS S AS S AS S AS S AS
F1 F1 F1 F1 F1 F1 F1 F1 F1 F1 F1 F1 F1 F1 F1 F1
R1 R1 R1 R1 F2 F2 F2 F2 R1 R1
Single D10A
Single H840A
Dual D10A
Dual H840A
Tandem D10A
Tandem H840A
Cas9
FokI-dCas9
TALEN

Supplementary Figure S9. Complete sets of assay results of HDR and NHEJ induced by sequence-specific nucleases in HeLa cells. (a–c) Genome-editing outcomes at *RBM20* (a), *GRN* (b), and *ATP7B* (c) in HeLa cells. HDR (red) and NHEJ (blue) allelic frequency (%) is shown. The results are shown in two different scales (highlighted in green). Conditions that gave equivalent or more HDR than NHEJ are highlighted by bold italic characters. Values are mean ± SEM. n = 6. The differences between the HDR and NHEJ frequencies were evaluated by Student's T-test (*, $p<0.05$). S, sense strand oligonucleotide donor; AS, antisense strand oligonucleotide donor. The background signals of the assays were subtracted (Supplementary Fig. S6).

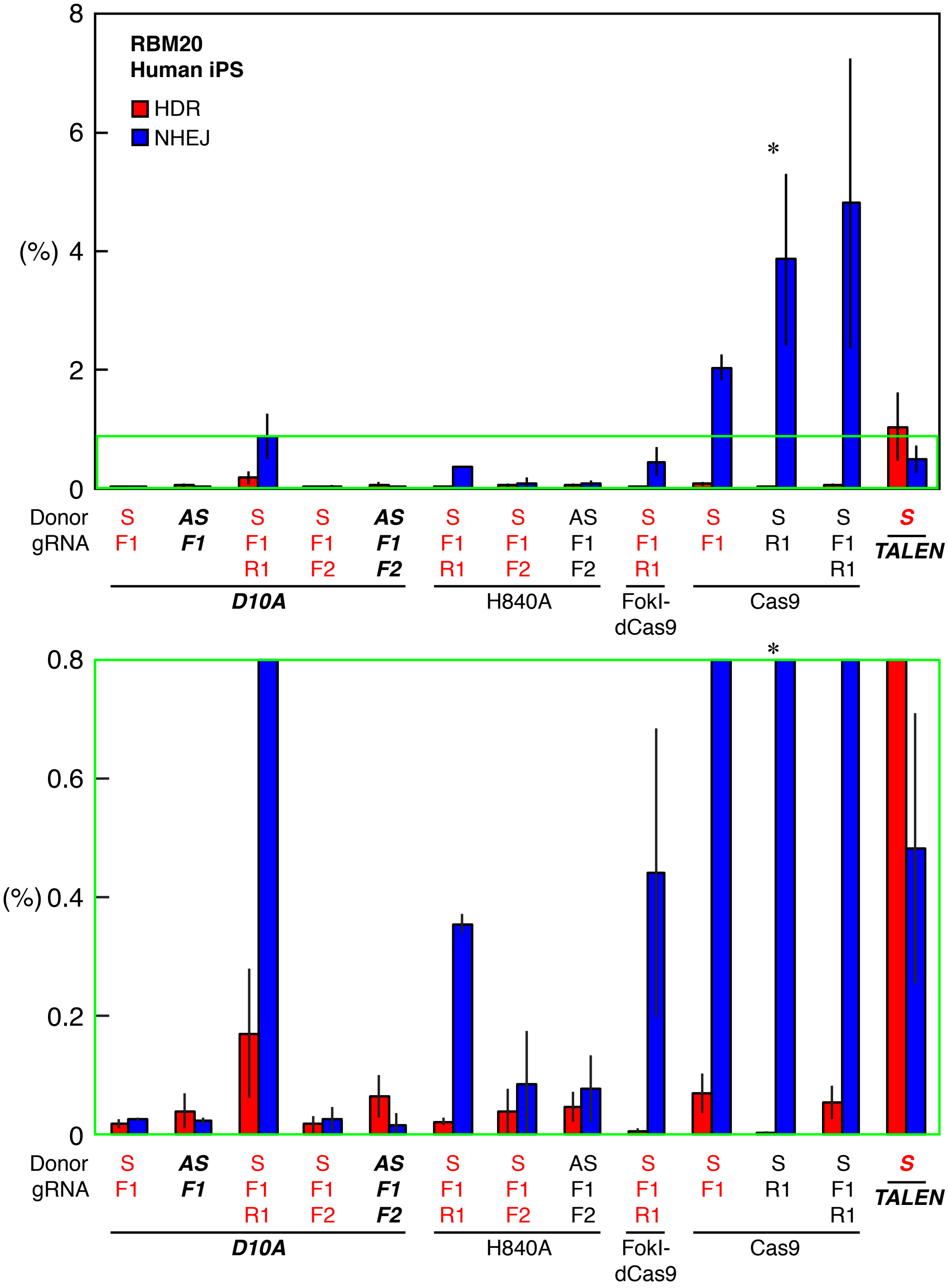

RBM20
Human iPS
HDR
NHEJ
(%)
0
2
4
6
8
0.2
0.4
0.6
0.8
Donor
gRNA
S F1
AS F1
S F1 R1
S F1 F2
AS F1 F2
S F1 R1
S F1 F2
AS F1 F2
S F1 R1
S F1
S R1
S F1 R1
S TALEN
D10A
H840A
FokI-dCas9
Cas9
*

Supplementary Figure S10. Measurement of HDR and NHEJ induced by sequence-specific nucleases in human iPSCs. Genome-editing outcomes of nucleases tested at *RBM20* in human iPSCs. HDR (red) and NHEJ (blue) allelic frequency (%) is shown. The results are shown in two different scales (highlighted in green). Conditions that gave equivalent or more HDR than NHEJ are highlighted in bold italic. Also, conditions summarized as a heat map in Fig. 6 are highlighted by red characters. TALENs induced 1% HDR and 0.5% NHEJ, but the Cas9 systems produced <0.2% HDR and similar or higher NHEJ levels compared to HDR. Values are mean ± SEM. n = 3. The differences between the HDR and NHEJ frequencies were evaluated by Student's T-test (*, $p<0.05$). S, sense strand oligonucleotide donor; AS, antisense strand oligonucleotide donor.

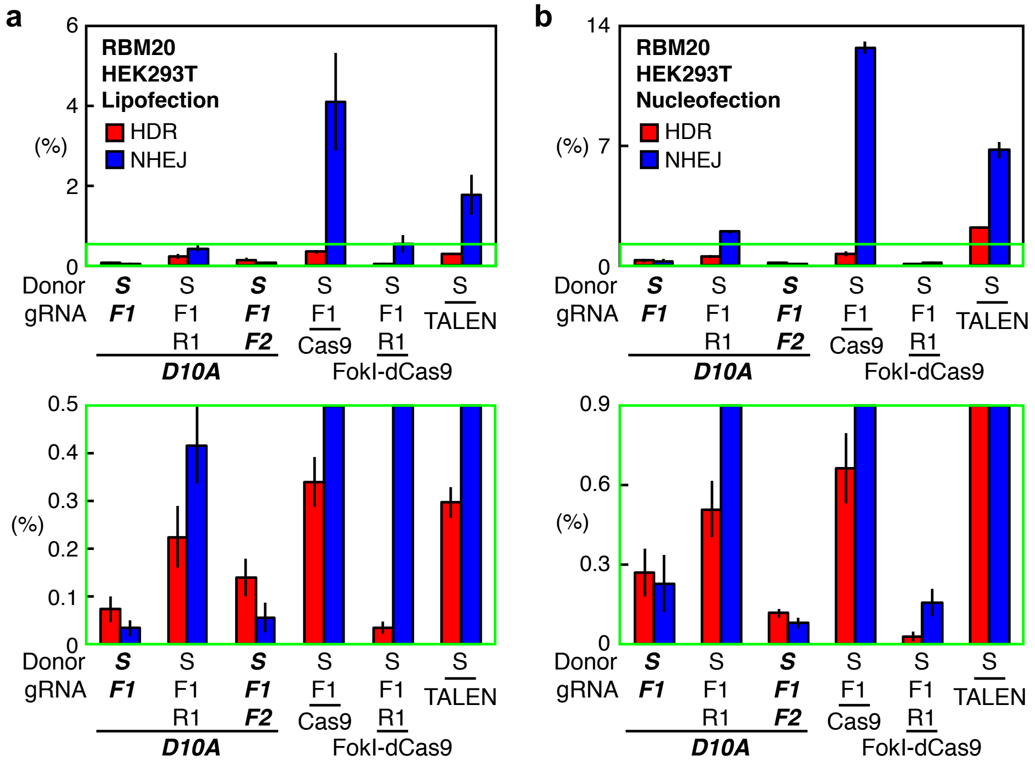
a
b
RBM20
HEK293T
Lipofection
Nucleofection
HDR
NHEJ
(%)
Donor
gRNA
S
F1
R1
F2
Cas9
TALEN
D10A
FokI-dCas9

Supplementary Figure S11. Comparison of different transfection methods in HEK293 cells. To address effects of different transfection methods (lipofection and electroporation) on genome-editing, we transfected HEK293 cells with indicated nucleases targeting RBM20 by using Nucleofector in the same way as for iPSCs (see Methods for details). HDR (red) and NHEJ (blue) allelic frequency (%) is shown. (a) Results of lipofection. Please note that the data shown here is a part of Supplementary Fig. S7. (b) Results of electroporation. Although electroporation generally gave higher genome-editing efficiency, the overall trend of the ratio of HDR and NHEJ is similar between the two different transfection methods. The results are shown in two different scales (highlighted in green). Conditions that gave equivalent or more HDR than NHEJ are highlighted by bold italic characters. Values are mean ± SEM. n = 6. The differences between the HDR and NHEJ frequencies were evaluated by Student's T-test (*, $p<0.05$). S, sense strand oligonucleotide donor; AS, antisense strand oligonucleotide donor. The background signals of the assays were subtracted (Supplementary Fig. S6).

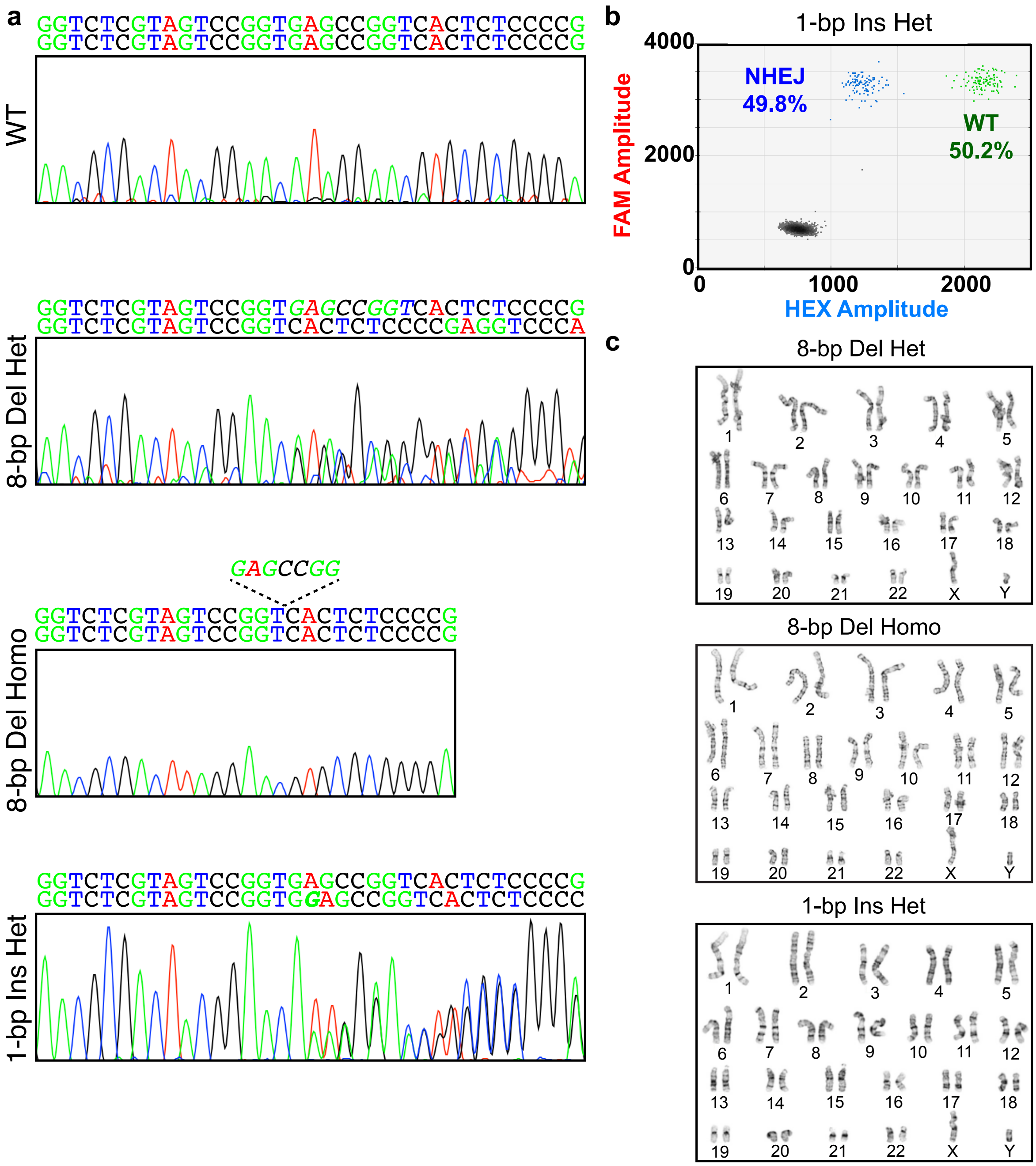
a
GGTCTCGTAGTCCGGTGAGCCGGTCACTCTCCCCG
GGTCTCGTAGTCCGGTGAGCCGGTCACTCTCCCCG
WT
GGTCTCGTAGTCCGGTGAGCCGGTCACTCTCCCCG
GGTCTCGTAGTCCGGTCACTCTCCCCGAGGTCCCA
8-bp Del Het
GAGCCGG
GGTCTCGTAGTCCGGTCACTCTCCCCG
GGTCTCGTAGTCCGGTCACTCTCCCCG
8-bp Del Homo
GGTCTCGTAGTCCGGTGAGCCGGTCACTCTCCCCG
GGTCTCGTAGTCCGGTGGAGCCGGTCACTCTCCCC
1-bp Ins Het
b
1-bp Ins Het
NHEJ
49.8%
WT
50.2%
FAM Amplitude
HEX Amplitude
4000
2000
0
0
1000
2000
c
8-bp Del Het
8-bp Del Homo
1-bp Ins Het
1
2
3
4
5
6
7
8
9
10
11
12
13
14
15
16
17
18
19
20
21
22
X
Y

Supplementary Figure S12. Isolation of iPSC lines with NHEJ in RBM20. By utilizing our ddPCR-based NHEJ detection assay and sib-selection (multiple rounds of serial dilution), we isolated iPSC line with a heterozygous or homozygous 8-bp deletion (8-bp Del Het or Homo), or heterozygous 1-bp insertion (1-bp Ins Het). (a) Sequence results of the isolated mutant iPSC lines. Sequences of the two alleles of the isolated iPSC lines are shown with the raw peaks. In 8-bp Del Het line, the 8-bp sequence deleted from the NHEJ allele is highlighted with italic in the wild-type allele. In 8-bp Del Homo line, the 8-bp sequence deleted from both alleles is highlighted with italic and an indication of its original location. In 1-bp Ins Het line, the single guanine insertion is highlighted with bold italic in the NHEJ allele. (b) Genotyping of the isolated iPSCs by ddPCR. The results for 1-bp Ins Het are shown as an example. The line showed 49.8% NHEJ and 50.2% WT allelic frequencies, precisely showing its heterozygous genotype. These results demonstrate that our ddPCR-based assay is accurate even with higher NHEJ frequencies. (c) Karyotype of the isolated RBM20 mutant iPSC lines. All the clones maintained a normal male karyotype. Each chromosome is annotated by its chromosomal number, X, or Y.

Supplementary Table S1 | Isolated iPSC clones and NHEJ events

Target gene	Nuclease used	Target modification by HDR	NHEJ event associated with HDR
RBM20	Dual Cas9-D10A	C-terminal 3x FLAG-Tag	34-bp insertion
GRN	Dual Cas9-D10A	Arg493Stop point mutation	302-bp deletion + 21-bp insertion
			24-bp deletion
			~100-bp deletion

bp, basepair

The sib-selection-based method was used to isolate iPSC clones that had desired HDR modifications and unwanted NHEJ events. All clones were generated with the dual Cas9-D10A system.

Supplementary Table S2 l gRNAs used in this study

	gRNA-F1	gRNA-R1	gRNA-F2	gRNA-R2
RBM20	GGTCTCGTAGTCCGGTGAGCCGG	CCATATCTGTGAGGGAGCCAAGG	AGATTCTAAATCCTGCTCCTTGG	GGGGAGAGTGACCGGCTCACCGG
GRN	GAAGGCTCGATCCTGCGAGAAGG	AGCCAGCCGGGCAGCAGTGCTGG	GGATCGCCAGCACTGCTGCCCGG	AGAGACCACTTCCTTCTCGCAGG
ATP7B	CATTGCCCTGGGCCGGTGGCTGG	GGCGTGTCGAAGAATGTCACAGG	CTGTGGCTGAGAAGGCGGAGAGG	TTGCCAAGTGTTCCAGCCACCGG

Point mutation sites are underlined.

Supplementary Table S3 | TALENs used in this study

	Forward TALEN<br>Target DNA sequence<br>(RVDs)	Reverse TALEN<br>Target DNA sequence<br>(RVDs)	Spacer DNA sequence
RBM20	TCACAGATATGGCCCAGA<br>(NG HD NI HD NI NN NI NG NI NG<br>NN NN HD HD HD NI NN NI)	TGACCGGCTCACCGGA<br>(NG NN NI HD HD NN NN HD NG HD<br>NI HD HD NN NN NI)	AAGGCCGCGGTCTCGTAG
GRN	TGCCCGGCTGGCTACA<br>(NG NN HD HD HD NN NN HD NG<br>NN NN HD NG NI HD NI)	TCCTTCTCGCAGGATCG<br>(NG HD HD NG NG HD NG HD NN<br>HD NI NN NN NI NG HD NN)	CCTGCAACGTGAAGGCT
ATP7B	TGTGTTCATTGCCCTGGG<br>(NG NN NG NN NG NG HD NI NG NG<br>NN HD HD HD NG NN NN NN)	TGCTGTTACCTTTGCCAA<br>(NG NN HD NG NN NG NG NI HD HD<br>NG NG NG NN HD HD NI NI)	CCGGTGGCTGGAACAC

Point mutation sites are underlined. Repeat variable di-residues (RVDs) used for these TALENs are also shown.

Supplementary Table S4 | Single-stranded oligonucleotide donor DNA used in this study

RBM20 sense	ACAGATATGGCCCAGAAAGGCCGCGGTCTAGTAGTCCGGTGAGCCGGTCACTCTCCCCGA
RBM20 antisense	TCGGGGAGAGTGACCGGCTCACCGGACTACTAGACCGCGGCCTTTCTGGGCCATATCTGT
GRN sense	CGGCTGGCTACACCTGCAACGTGAAGGCTTGATCCTGCGAGAAGGAAGTGGTCTCTGCCC
GRN antisense	GGGCAGAGACCACTTCCTTCTCGCAGGATCAAGCCTTCACGTTGCAGGTGTAGCCAGCCG
ATP7B sense	CATGCTCTTTGTGTTCATTGCCCTGGGCCTGTGGCTGGAACACTTGGCAAAGGTAACAGC
ATP7B antisense	GCTGTTACCTTTGCCAAGTGTTCCAGCCACAGGCCCAGGGCAATGAACACAAAGAGCATG

Point mutation sites are underlined.

Supplementary Table S5 | Probe-primer sets used in this study

	Sequence	Fluor-Quencher	Final Concentration (1x)
RBM20 Assay Components			
Primers			
Forward	CTGTGTGTGGGTGGGGT		900 nM
Reverse	AGGAGGTGAAGCTGGGAG		900 nM
Probes			
Reference	TGGGAGGTGTGAAGATTCTAAATC	FAM-Zen	250 nM
HDR	CCGCGGTCTAGTAGTCC	FAM-Zen	250 nM
Dark	CCGCGGTCTCGTAGTCC	None. Add 3' phosphate	500 nM
gRNA F2/R1 NHEJ	TGCTCCTTGGCTCCCT	HEX-Zen	250 nM
FokI-dCas9 NHEJ	CCTTTCTGGGCCATATCTGTG	HEX *(no Zen)*	250 nM
gRNA F1/R2 NHEJ	AGAGTGACCGGCTCAC	HEX-Zen	250 nM
TALEN NHEJ	AGGCCGCGGTCTCGT	HEX-Zen	250 nM
GRN Assay Components			
Primers			
Forward	CTGGATAGGGGAGCTAAG		900 nM
Reverse	GTCTGGTTATCATGGCAG		900 nM
Probes			
Reference	CAGGAACATAATGCCATTCTGTGC	FAM-Zen	250 nM
HDR	CAGGATCAAGCCTTCACG	FAM-Zen	250 nM
Dark	CAGGATCGAGCCTTCACG	None. Add 3' phosphate	500 nM
gRNA F2/R1 NHEJ	TCGCCAGCACTGCTGC	HEX-Zen	250 nM
FokI-dCas9 NHEJ	TGGCTACACCTGCAACG	HEX-Zen	250 nM
gRNA F1/R2 NHEJ	CTGCGAGAAGGAAGTGGT	HEX-Zen	250 nM

TALEN NHEJ	TCGAGCCTTCACGTTGCA	HEX-Zen	250 nM
ATP7B Assay Components			
Primers			
Forward	TGCTTATGTTTATTCTCTGGTCATC		900 nM
Reverse	CCTGAAGCTGCTGTTACCTT		900 nM
Probes			
Reference	TGGTGGTTGCTGTGGCT	FAM-Zen	250 nM
HDR	TGGGCC<u>T</u>GTGGCTG	FAM-Zen	250 nM
Dark	CTGGGCC<u>G</u>GTGGCT	None. Add 3' phosphate	500 nM
gRNA R1 NHEJ	CCCTGTGACATTCTTCGAC	HEX-Zen	250 nM
gRNA F2 NHEJ	AGGCGGAGAGGAGCCC	HEX-Zen	250 nM
FokI-dCas9 NHEJ	CATGCTCTTTGTGTTCATTGC	HEX-Zen	250 nM
gRNA F1/R2 NHEJ	CTGGGCCGGTGGCTG	HEX-Zen	250 nM
TALEN NHEJ	CCGGTGGCTGGAACACT	HEX-Zen	250 nM

Point mutations are underlined.

Supplementary Table S6 | NHEJ and dark probe sets used in this study

RBM20/GRN probe sets	Dark	gRNA F2/R1 NHEJ	FokI-dCas9 NHEJ	gRNA F1/R2 NHEJ	TALEN NHEJ
Single Cas9 assay (F1/R2)	+	-	-	+	-
Single Cas9 assay (F2/R1)	+	+	-	-	-
Dual Cas9 assay (F1/R2+F2/R1)	+	+	-	+	-
FokI-dCas9 assay	+	-	+	-	-
TALEN assay	-	-	-	-	+

ATP7B probe sets	Dark	gRNA R1 NHEJ	gRNA F2 NHEJ	FokI-dCas9 NHEJ	gRNA F1/R2 NHEJ	TALEN NHEJ
Single Cas9 assay (R1)	+	+	-	-	-	-
Single Cas9 assay (F2)	+	-	+	-	-	-
Single Cas9 assay (F1/R2)	-	-	-	-	+	-
Dual Cas9 assay (F1/R2+R1)	-	+	-	-	+	-
Dual Cas9 assay (F1/R2+F2)	-	-	+	-	+	-
FokI-dCas9 assay	+	-	-	+	-	-
TALEN assay	-	-	-	-	-	+

All assays also contain a reference probe and HDR probe (not included in table).

Supplementary Table S7 | Synthesized DNA of HDR and NHEJ alleles used in validation of the RBM20 assays

RBM20 HDR	GTCCTCTGCACGGAAGCCAGAAGGGAGGAAAAGGCTTTCTCCTGAACCACTCTGTGTGGTTCTGTAGAGTTGGGAGTTAAGAGTGTACACAGTTA<br>CATGCACAGTATATCTAAGACAGAGACTGTGTGTCTGTGTGTGGGTGGGGTGGGATGGGAGGTGTGAAGATTCTAAATCCTGCTCCTTGGCTCC<br>CTCACAGATATGGCCCAGAAAGGCCGCGGTCT**A**GTAGTCCGGTGAGCCGGTCACTCTCCCCGAGGTCCCACACTCCCAGCTTCACCTCCTGCA<br>GCTCTTCCCACAGCCCTCCGGGCCCCTCCCGGGCTGACTGGGGCAATGGCCGGGACTCCTGGGAGCACTCTCCCTATGCCAGGAGGGAGGAA<br>GAGCGAGACCCGGCTCCCTGGAGGGACAACGGAGATGACAAGAGGGACAGGATGGACCCCTGGGCACATGATCGCAAACACCACCC
RBM20 F2/R1 1-bp deletion	GTCCTCTGCACGGAAGCCAGAAGGGAGGAAAAGGCTTTCTCCTGAACCACTCTGTGTGGTTCTGTAGAGTTGGGAGTTAAGAGTGTACACAGTTA<br>CATGCACAGTATATCTAAGACAGAGACTGTGTGTCTGTGTGTGGGTGGGGTGGGATGGGAGGTGTGAAGATTCTAAATCCTGCTCCTTG–CTCCC<br>TCACAGATATGGCCCAGAAAGGCCGCGGTCTCGTAGTCCGGTGAGCCGGTCACTCTCCCCGAGGTCCCACACTCCCAGCTTCACCTCCTGCAG<br>CTCTTCCCACAGCCCTCCGGGCCCCTCCCGGGCTGACTGGGGCAATGGCCGGGACTCCTGGGAGCACTCTCCCTATGCCAGGAGGGAGGAAG<br>AGCGAGACCCGGCTCCCTGGAGGGACAACGGAGATGACAAGAGGGACAGGATGGACCCCTGGGCACATGATCGCAAACACCACCC
RBM20 FokI-dCas9 1-bp deletion	GTCCTCTGCACGGAAGCCAGAAGGGAGGAAAAGGCTTTCTCCTGAACCACTCTGTGTGGTTCTGTAGAGTTGGGAGTTAAGAGTGTACACAGTTA<br>CATGCACAGTATATCTAAGACAGAGACTGTGTGTCTGTGTGTGGGTGGGGTGGGATGGGAGGTGTGAAGATTCTAAATCCTGCTCCTTGGCTCC<br>CTCACAGATATGGCCCAGAA–GGCCGCGGTCTCGTAGTCCGGTGAGCCGGTCACTCTCCCCGAGGTCCCACACTCCCAGCTTCACCTCCTGCAG<br>CTCTTCCCACAGCCCTCCGGGCCCCTCCCGGGCTGACTGGGGCAATGGCCGGGACTCCTGGGAGCACTCTCCCTATGCCAGGAGGGAGGAAG<br>AGCGAGACCCGGCTCCCTGGAGGGACAACGGAGATGACAAGAGGGACAGGATGGACCCCTGGGCACATGATCGCAAACACCACCC
RBM20 FokI-dCas9 1-bp insertion	GTCCTCTGCACGGAAGCCAGAAGGGAGGAAAAGGCTTTCTCCTGAACCACTCTGTGTGGTTCTGTAGAGTTGGGAGTTAAGAGTGTACACAGTTA<br>CATGCACAGTATATCTAAGACAGAGACTGTGTGTCTGTGTGTGGGTGGGGTGGGATGGGAGGTGTGAAGATTCTAAATCCTGCTCCTTGGCTCC<br>CTCACAGATATGGCCCAGAA***T***AGGCCGCGGTCTCGTAGTCCGGTGAGCCGGTCACTCTCCCCGAGGTCCCACACTCCCAGCTTCACCTCCTGCA<br>GCTCTTCCCACAGCCCTCCGGGCCCCTCCCGGGCTGACTGGGGCAATGGCCGGGACTCCTGGGAGCACTCTCCCTATGCCAGGAGGGAGGAA<br>GAGCGAGACCCGGCTCCCTGGAGGGACAACGGAGATGACAAGAGGGACAGGATGGACCCCTGGGCACATGATCGCAAACACCACCC

RBM20 FokI-dCas9<br>4-bp insertion	GTCCTCTGCACGGAAGCCAGAAGGGAGGAAAAGGCTTTCTCCTGAACCACTCTGTGTGGTTCTGTAGAGTTGGGAGTTAAGAGTGTACACAGTTA<br>CATGCACAGTATATCTAAGACAGAGACTGTGTGTCTGTGTGTGGGTGGGGTGGGATGGGAGGTGTGAAGATTCTAAATCCTGCTCCTTGGCTCC<br>CTCACAGATATGGCCCAGAA***TGAC***AGGCCGCGGTCTCGTAGTCCGGTGAGCCGGTCACTCTCCCCGAGGTCCCACACTCCCAGCTTCACCTCCT<br>GCAGCTCTTCCCACAGCCCTCCGGGCCCCTCCCGGGCTGACTGGGGCAATGGCCGGGACTCCTGGGAGCACTCTCCCTATGCCAGGAGGGAG<br>GAAGAGCGAGACCCGGCTCCCTGGAGGGACAACGGAGATGACAAGAGGGACAGGATGGACCCCTGGGCACATGATCGCAAACACCACCC
RBM20 TALEN<br>NHEJ	GTCCTCTGCACGGAAGCCAGAAGGGAGGAAAAGGCTTTCTCCTGAACCACTCTGTGTGGTTCTGTAGAGTTGGGAGTTAAGAGTGTACACAGTTA<br>CATGCACAGTATATCTAAGACAGAGACTGTGTGTCTGTGTGTGGGTGGGGTGGGATGGGAGGTGTGAAGATTCTAAATCCTGCTCCTTGGCTCC<br>CTCACAGATATGGCCCAGAAAGGCCGCG**-**TCTCGTAGTCCGGTGAGCCGGTCACTCTCCCCGAGGTCCCACACTCCCAGCTTCACCTCCTGCAG<br>CTCTTCCCACAGCCCTCCGGGCCCCTCCCGGGCTGACTGGGGCAATGGCCGGGACTCCTGGGAGCACTCTCCCTATGCCAGGAGGGAGGAAG<br>AGCGAGACCCGGCTCCCTGGAGGGACAACGGAGATGACAAGAGGGACAGGATGGACCCCTGGGCACATGATCGCAAACACCACCC
RBM20 F1/R2<br>NHEJ	GTCCTCTGCACGGAAGCCAGAAGGGAGGAAAAGGCTTTCTCCTGAACCACTCTGTGTGGTTCTGTAGAGTTGGGAGTTAAGAGTGTACACAGTTA<br>CATGCACAGTATATCTAAGACAGAGACTGTGTGTCTGTGTGTGGGTGGGGTGGGATGGGAGGTGTGAAGATTCTAAATCCTGCTCCTTGGCTCC<br>CTCACAGATATGGCCCAGAAAGGCCGCGGTCTCGTAGTCCGGT**-**AGCCGGTCACTCTCCCCGAGGTCCCACACTCCCAGCTTCACCTCCTGCAG<br>CTCTTCCCACAGCCCTCCGGGCCCCTCCCGGGCTGACTGGGGCAATGGCCGGGACTCCTGGGAGCACTCTCCCTATGCCAGGAGGGAGGAAG<br>AGCGAGACCCGGCTCCCTGGAGGGACAACGGAGATGACAAGAGGGACAGGATGGACCCCTGGGCACATGATCGCAAACACCACCC

Introduced changes are all bold. The C>A substitutions introduced by HDR are underlined. Deletions are designated by hyphens. Insertions are italic.

Supplementary Table S8 | Genes and mutations engineered in this study

Gene symbol	Name	Nucleotide	Amino Acid	Reference
RBM20	RNA binding motif protein 20	1906C>A	Arg636Ser	Brauch et al.[1]
GRN	Progranulin	1477C>T	Arg493Stop	Chen-Plotkin et al.[2]
ATP7B	ATPase, Cu++ transporting, beta polypeptide	2333G>T	Arg778Leu	Ferenci et al.[3]

References

1 Brauch, K. M. *et al.* Mutations in ribonucleic acid binding protein gene cause familial dilated cardiomyopathy. *J Am Coll Cardiol* **54**, 930-941 (2009).

2 Chen-Plotkin, A. S. *et al.* Genetic and clinical features of progranulin-associated frontotemporal lobar degeneration. *Arch Neurol* **68**, 488-497 (2011).

3 Ferenci, P. Regional distribution of mutations of the ATP7B gene in patients with Wilson disease: impact on genetic testing. *Hum Genet* **120**, 151-159 (2006).
